# Effects of the aldehyde-derived ring substituent on the properties of two new bioinspired trimethoxybenzoylhydrazones: methyl vs nitro groups

**DOI:** 10.3762/bjoc.19.125

**Published:** 2023-11-10

**Authors:** Dayanne Martins, Roberta Lamosa, Talis Uelisson da Silva, Carolina B P Ligiero, Sérgio de Paula Machado, Daphne S Cukierman, Nicolás A Rey

**Affiliations:** 1 Departmento de Química, Pontifícia Universidade Católica do Rio de Janeiro (PUC-Rio), Rio de Janeiro, 22451-900, Brazil; 2 Instituto de Química, Universidade Federal Rural do Rio de Janeiro (UFRRJ), Seropédica 23890-000, Brazil; 3 Departamento de Química Inorgânica, Universidade Federal Fluminense (UFF), Niterói, 24020-141, Brazil; 4 Instituto de Química, Universidade Federal do Rio de Janeiro (UFRJ), Rio de Janeiro 21945-970, Brazil; 5 Departamento de Química Geral e Inorgânica, Universidade do Estado do Rio de Janeiro (UERJ), Rio de Janeiro, 20950-000, Brazil

**Keywords:** DFT calculations, *N*-acylhydrazones, phenol acidity, ring substituents, XRD

## Abstract

*N*-Acylhydrazones are a versatile class of organic compounds with a diversity of potential applications. In this study, two new structure-related 3,4,5-trimethoxybenzoyl-containing *N*-acylhydrazones were synthesized and fully characterized, both in solution and in the solid state. The compounds differ with respect to the carbonyl precursors, i.e., 3-substituted salicylaldehydes with either a methyl or a nitro group. Single crystals of both compounds were isolated from the respective mother liquors and, in both cases, XRD confirmed the obtention of the (*E*)-isomer, in an *anti*-conformation. Computational calculations (gas and water phases) were performed in order to confirm some of the structural and vibrational aspects of the compounds. An important intramolecular H bond involving the phenolic hydroxy group and the azomethine nitrogen was identified in the solid state and seems to be maintained in solution. Moreover, the presence of the electron-withdrawing nitro substituent makes this interaction stronger. However, the contact should probably not subsist for the nitro compound under physiological conditions since the presence of this substituent significantly affects the p*K*_a_ of the phenol: an apparent value of 5.68 ± 0.02 was obtained. This also impacts the basicity of the azomethine nitrogen and, as a consequence, increases the hydrazone’s susceptibility to hydrolysis. Nevertheless, both compounds are stable at physiological-like conditions, especially the methyl-derived one, which qualifies them for further toxicological and activity studies, such as those involving trivalent metal ions sequestering in the context of neurodegenerative diseases.

## Introduction

*N*-Acylhydrazones are a class of compounds that contain the hydrazonic functional group (–NH–N=C–) attached to an acyl group, which can be modified to generate a range of different structures with varying properties [1]. The versatility of this class of compounds is also related to the ability of *N*-acylhydrazones to exist as different isomers and/or tautomers. They can exist as geometric isomers (*E*/*Z*), which differ in the orientation of the groups around the carbon–nitrogen double bond [2–3], as well as amido and iminol tautomers [4]. The ability to undergo *E*/*Z* isomerization in a stimuli-responsive imine bond is what makes this class useful for applications in the field of molecular electronics, as switchers [5–6]. In addition, these compounds can also adopt *syn*- or *antiperiplanar* conformations, due to the constriction of the rotation around the conjugated amide single bond (N–C=O).

*N*-Acylhydrazones have also gained attention in literature due to other applications, ranging from medicine to supramolecular chemistry [7–8]. Among their applicability is the area of optoelectronic devices, in which they are used for the manufacture of organic light-emitting diodes (OLEDs) [9–11]. Moreover, studies involving *N*-acylhydrazonic derivatives have highlighted their suitability for the treatment of pathologies associated with infection and/or inflammation [12–17]. Antimicrobial activity is one of the most frequently studied and reported biological properties of this class [2,18–20]. Angelova and co-workers, for example, reported the ability of sulfonyl hydrazones and 4-methyl-1,2,3-thiadiazole-based hydrazone derivatives to inhibit the growth of several bacterial strains by interfering with their metabolism or cell membrane integrity [21].

In the context of cancer therapy development, metal complexes of *N*-acylhydrazones stand out. For example, Firmino et al. demonstrated that gallium(III) complexes of isoniazid-derived hydrazones exhibit strong cytotoxicity against HL-60 and HCT-116 cancer cell lines [22]. The study also found that those coordination compounds were selective towards abnormal cells, exhibiting lower toxicity for healthy human hepatocytes. On the other hand, an important development in cancer research is the use of physiological metal ion complexes, which afford more biocompatibility and thus less side-effects in therapy [23]. In this sense, we have reported dicopper(II) complexes from different *N*-acylhydrazonic binucleating ligands with potent antiproliferative activity against a panel of cancer cell lines [24–26].

On the field of neurodegeneration, our research group was the first to establish the suitability of *N*-acylhydrazones as novel metallophores able to affect protein aggregation and/or oxidation enhanced by physiological metal–protein anomalous interactions related to Alzheimer's (AD) and Parkinson's (PD), as well as to prion diseases [27–36]. Our lead compound INHHQ (or 8-hydroxyquinoline-2-carboxaldehyde isonicotinoyl hydrazone) has been successfully tested in the prevention of short- and long-term memory deficits in a mice model of sporadic AD [33]. Additionally, INHHQ decreases copper-mediated production of reactive oxygen species (ROS) in vitro, which may be another mechanism through which the compound exerts its protective effects in the brain.

From a drug development perspective, however, INHHQ has some pharmacological limitations, such as low solubility and certain susceptibility to hydrolysis in a water-rich medium. The protective character and high metallophoric potential of INHHQ, nevertheless, prompted us to design and synthesize new optimized derivatives. In this context, we recently described a family of pharmocologically improved *N*-acylhydrazones containing the 1-methylimidazole moiety [37]. In 2020, we proved the promising anti-PD and metallophoric effect, especially towards intracellularly relevant copper(I) ions, of X1INH (1-methyl-1*H*-imidazole-2-carboxaldehyde isonicotinoyl hydrazone) [32].

This year, we evaluated the effects of the presence of three methoxy substituents in an *N*-acylhydrazone derived from 3,4,5-trimethoxybenzoic acid hydrazide, a modification inspired by mescaline, the active principle of the hallucinogenic cactus peyote, which could result in a greater BBB penetration [36]. In this study, however, the structural modifications in the compound did not seem to significantly affect its pharmacological properties and metallophoric potential against copper(II) when compared to the unsubstituted counterpart. Nevertheless, the bioinspired compound was still able to reduce oxidative stress and affect the aggregation of the amyloid-β peptide, related to pathophysiological events in AD.

**Scheme 1:**
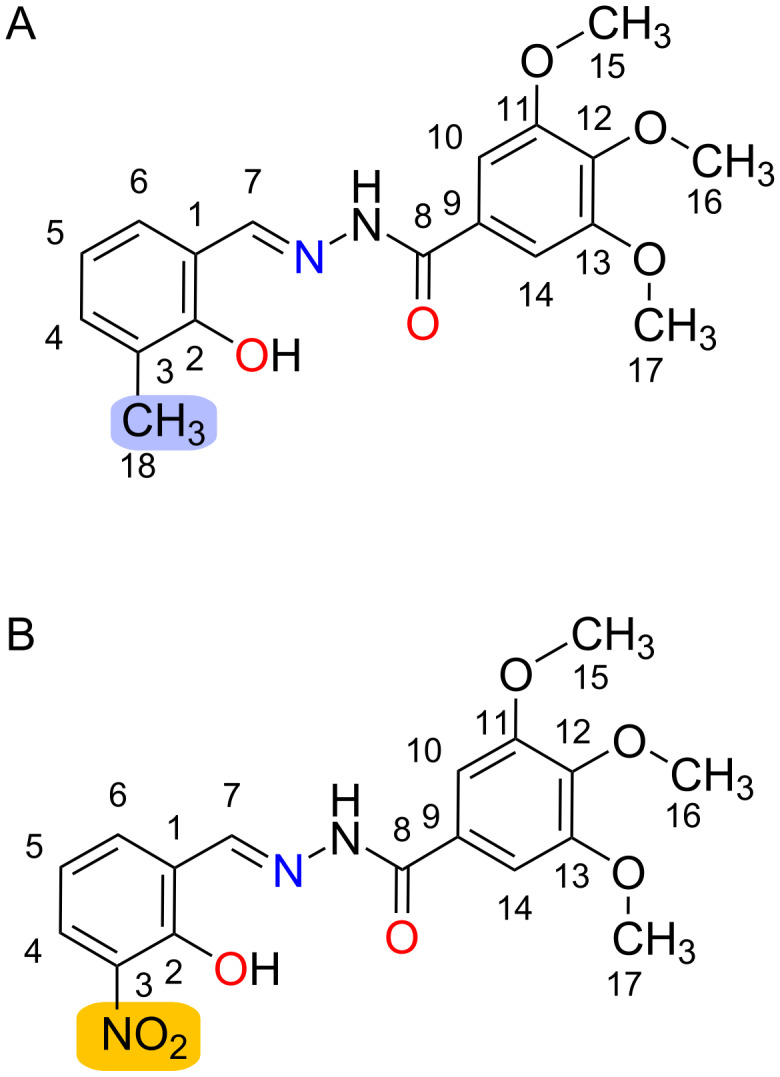
Structure of (A) 3-methylsalicylaldehyde 3,4,5-trimethoxybenzoyl hydrazone (**hdz-CH_3_**) and (B) 3-nitrosalicylaldehyde 3,4,5-trimethoxybenzoyl hydrazone (**hdz-NO_2_**).

As a continuation of our long-term effort on the development of potentially bioactive *N*-acylhydrazones, the present work comprises a structural and spectroscopic comparison, from both experimental and theoretical viewpoints, of two structure-related hydrazones. Both compounds are new, and derived from the same 3,4,5-trimethoxybenzoic acid hydrazide, but differ with respect to the carbonyl precursors: herein, 3-substituted salicylaldehydes (Scheme 1) are used, which assure for a harder donor-atoms set in order to target trivalent metal ions such as aluminum(III), which has been proposed to display a role in neurodegeneration [38].

A comparative study between these two *N*-acylhydrazones is interesting, especially considering that they possess different substituents at the same position in the phenol ring: the electron-donating methyl group (**hdz-CH_3_**) and the electron-withdrawing nitro group (**hdz-NO_2_**). It is expected that those substituents impact the chemical (e.g., acidity and hydrolysis susceptibility) as well as the structural and spectroscopic properties of the compounds.

## Results and Discussion

The methyl-substituted **hdz-CH_3_** and its nitro-containing analogue **hdz-NO_2_** were isolated as beige and light-yellow solids with 78% and 44% yield, respectively. Thermal analyses between 25 and 350 °C were performed in order to verify the hydration status of the bulk. Regarding **hdz-CH_3_**, a weight loss of 9.78% from around 80 to 190 °C was observed, suggesting the presence of two crystallization water molecules in the network (calcd.: 9.47% for C_18_H_20_O_5_N_2_·2H_2_O, MW = 380.39 g mol^−1^). On the other hand, **hdz-NO_2_** did not show any mass loss below 250 °C, indicating the absence of solvation molecules in the sample (C_17_H_17_O_7_N_3_, MW = 375.34 g mol^−1^).

**Figure 1:**
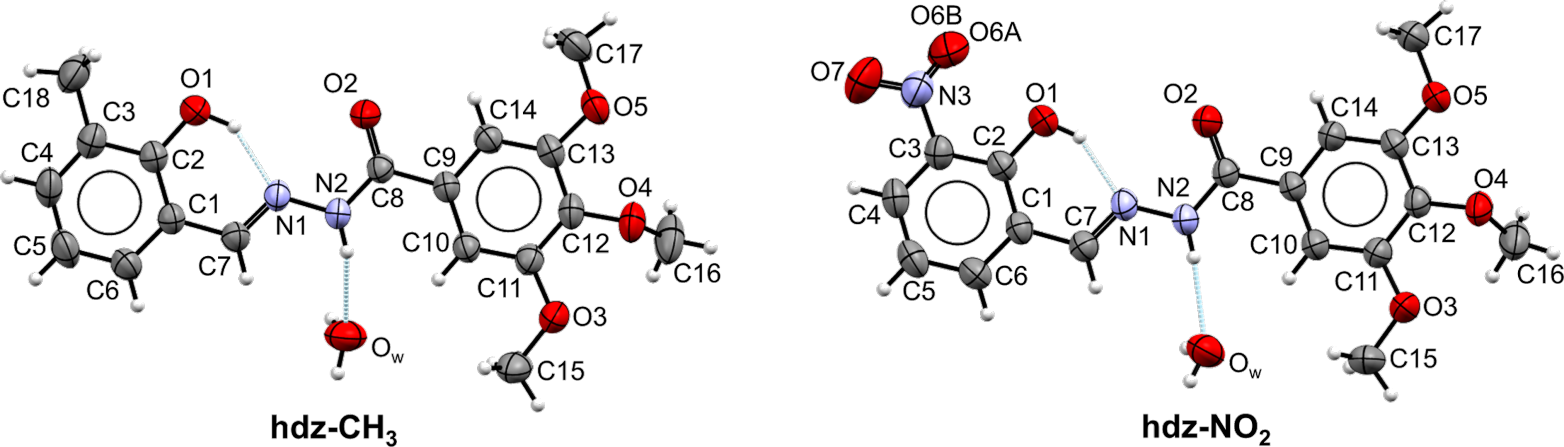
ORTEP representation of the new *N*-acylhydrazones synthesized in the present work, drawn with thermal ellipsoids at 30% probability. Left: **hdz-CH_3_** and right: **hdz-NO_2_**.

Single crystals of both compounds, as monohydrates, were isolated from the respective mother liquors. The structures of **hdz-CH_3_** and **hdz-NO_2_** are displayed in Figure 1 and an overview of the crystallographic data can be found in Table 1.

**Table 1:** Crystal, data collection and refinement parameters for **hdz-CH_3_** and **hdz-NO_2_**.^a^

Data	**hdz-CH** **_3_**	**hdz-NO** **_2_**

crystal size (mm)	0.99 × 0.14 × 0.14	0.57 × 0.32 × 0.12
empirical formula	C_18_H_22_N_2_O_6_	C_17_H_19_N_3_O_8_
formula weight (g mol^−1^)	362.37	393.35
F(000)	1536	412
temperature (K)	293	293
absorption coefficient μ (mm^−1^)	0.101	0.117
calculated density (g·cm^−3^)	1.337	1.452
crystal system	monoclinic	triclinic
space group	*C*_2_/*c* (n° 15)	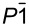 (n° 2)
*a*, *b*, *c* (Å)	32.1401(15) 8.4828(4) 14.3708(6)	8.3230(9) 8.5243(9) 13.3392(14)
α, β, γ (°)	90.000 113.2650(10) 90.000	88.593(4) 87.468(4) 72.105(3)
cell volume (Å^3^)	3599.4(3)	899.66(17)
*Z, Z’*	8, 1	2, 1
reflections collected, *R*_int_	48639, 0.0363	35549, 0.1407
independent reflections	3651	3238
index ranges	−40 < *h* < 40 −10 < *k* < 10 −17 < *l* < 17	−10 < *h* < 10 −10 < *k* < 10 −16 < *l* < 16
data/restrains/parameters	3651/3/250	3238/9/277
final residual factor [*I* > 2σ(I)]	R_1_: 0.0486 wR_2_: 0.1450	R_1_: 0.0746 wR_2_: 0.1929
goodness-of-fit on *F^2^*	1.11	0.99
T_min_, T_max_	0.906, 0.986	0.936, 0.986
theta range for data collection (°)	2.789 to 26.372	2.573 to 25.345
largest diff. peak and hole (e·Å^−3^)	0.17, −0.19	0.290, −0.291

^a^*a*, *b*, *c*, α, β, γ*:* unit cell parameters; *Z*: formula unit per unit cell; *Z’*: number of formula units in the crystallographic unit cell divided by the number of independent general positions; F(000): structure factor in the zeroth-order case; F: structure factor; F^2^: squared structure factor; T: transmission factor.

**Figure 2:**
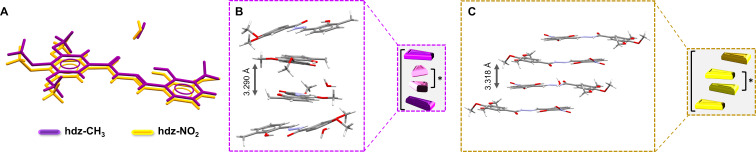
(A) Superposition of molecular structures and stacked motifs of (B) **hdz-CH_3_** and (**C**) **hdz-NO_2_**.

Both molecules are near planar and correspond to the (*E*)-isomer, in an *antiperiplanar* conformation. Superposition of the structures (Figure 2A) shows that spatial arrangements are nearly the same and even the crystallization water molecules were allocated in nearby sites, interacting as H-acceptors in a hydrogen bond with the respective N2H groups. In spite of these similarities, the compounds were indexed in different space groups. The methyl-containing **hdz-CH_3_** crystallized in the monoclinic system, *C*2/*c* space group, while the nitro derivative **hdz-NO_2_** belongs to the 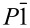 group, from the triclinic system. In both cases, a moderate to strong intramolecular H-bond involving the phenol oxygen O1 as H-donor and the azomethine nitrogen N1 as H-acceptor is observed, which originates six-membered cyclic motifs with a graph-set 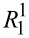 (6) [39]. The hydrogen atom is closer to O1 than to N1, indicating that the preferred protonation site is the former, as can be observed at the Fourier difference maps (Figure S1A and S1B in Supporting Information File 1), but the presence of the hydrogen bond influences the contiguous aromatic system, as can be inferred by the characteristic elongation of the C1–C2 bond, which is 1.405(2) Å in **hdz-CH_3_** and 1.411(3) Å in **hdz-NO_2_** [40–42]. The effect is also noticed through the HOMA (harmonic oscillator model of aromaticity) indexes [43] of the rings: while the trimethoxy-substituted, hydrazide-derived one presents values of 0.995 (**hdz-CH_3_**) and 0.990 (**hdz-NO_2_**), indicating high aromaticity, the aldehyde-derived ring shows HOMA indexes of 0.964 (**hdz-CH_3_**) and 0.961 (**hdz-NO_2_**), suggesting that, for both compounds, this H-bond decreases the aromaticity of the phenol-containing ring. Finally, O1···N1 distances are 2.581(2) and 2.539(3) Å for **hdz-CH_3_** and **hdz-NO_2_**, respectively (Table 2). Thus, all the structural evidence discussed above converges to the conclusion that the presence of the electron-withdrawing nitro substituent in **hdz-NO_2_** makes the intramolecular H-bond stronger.

**Table 2:** H-bonding parameters for **hdz-CH_3_** and **hdz-NO_2_**.

D–H···A	Symmetry operation	D–H (Å)	H···A (Å)	D···A (Å)	D–H···A (°)	% Σ*vdWr*

**hdz-CH** **_3_**						

O1–H1···N1	intra	0.93(2)	1.772(18)	2.581(2)	144(2)	64.4
Ow–Hw1···O2	–	0.84(3)	2.00(3)	2.816(2)	166(3)	73.5
N2–H2a···Ow	1−x,1−y,1−z	0.840(17)	2.164(18)	2.910(2)	148(2)	79.6
C14–H14···O2	intra	0.9300	2.400	2.734(2)	101.00	87.3

**hdz-NO** **_2_** ^a^						

O1–H1···N1	intra	0.86(4)	1.79(3)	2.539(3)	146(3)	65.1
Ow–Hw2···O4	–	0.83(5)	2.03(5)	2.839(4)	169(5)	74.6
Ow–Hw1···O2A	1−*x* , −*y*, 1−*z*	0.82(4)	2.48(5)	3.143(13)	138(4)	91.2
Ow–Hw1···O1	1−*x* , −*y* , 1−*z*	0.82(4)	2.34(4)	3.061(3)	147(4)	86.0
N2–H2a···Ow	1−*x* , 1−*y* , 1−*z*	0.83(2)	2.09(2)	2.898(4)	163(3)	76.8
C5–H5···O2	−1+*x* , *y* , 1+*z*	0.9300	2.5800	3.175(4)	162.00	94.8
C10–H10···Ow	1−*x* , 1−*y* , 1−*z*	0.9300	2.4600	3.360(4)	162.00	91.2
C14–H14···O4	intra	0.9300	2.4400	7.758(4)	100.00	89.7
C16–H16c···O5	intra	0.9600	2.4100	2.975(5)	117.00	88.6
C17–H17b···O3	1−*x* , −*y* , 1−*z*	0.9600	2.5900	3.536(5)	168.00	95.2

^a^Data shown only for the major component of disorder.

Regarding lattice organization, both structures exhibit stacked motifs. In **hdz-CH_3_**, the 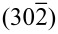 planes were organized by C–H···O non-conventional H-bonds comprising the methoxy groups (Figure 2B). In the columns, there are antiparallel dimers (marked as *) sandwiched between two non-parallel molecules. The planes in **hdz-NO_2_** present similar intermolecular interactions, but they grow as (101) planes. The columns also present the antiparallel dimers, but they are intercalated by antiparallel slipped molecules (Figure 2C).

The supramolecular organization of the *N*-acylhydrazones was also studied through their Hirshfeld surfaces, in which π–π interactions play an important role. When the normalized distance between a molecule and the closest neighbors are plotted (Figures S2A and S2C, and S3A and S3C in Supporting Information File 1), it becomes possible to calculate the contribution of each type of intermolecular interaction to the whole profile [44]. In our case, results show that the sum of π···π and C–H···π contacts comprises almost 20% of the Hirshfeld surface for both compounds. The curvedness maps were also plotted over the Hirshfeld surfaces and evidence the strong flatness of the structures (Figures S2B and S3B in Supporting Information File 1). Important contributions of hydrogen bonds (O···H/H···O) can also be found, especially for **hdz-NO_2_**. Besides the interaction maps, crystal structures themselves may be used to calculate the electrostatic potential over an electronic density map as well. For **hdz-NO_2_**, results show the alternated slipped stack motif involves the assembly of portions with opposite electrostatic potentials.

**Figure 3:**
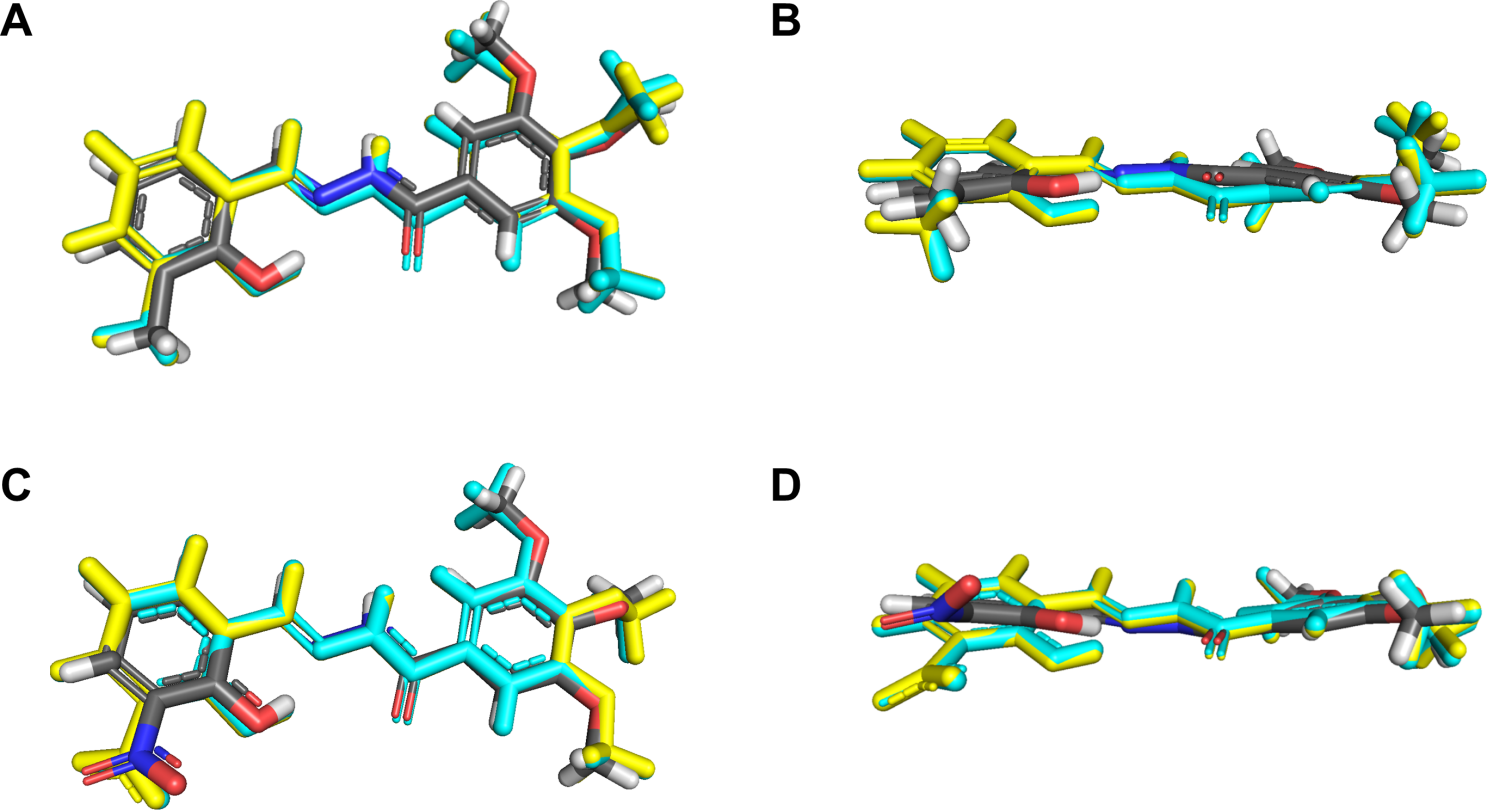
Overlap of the experimental (carbon atoms colored in gray) and theoretical structures (calculated with B3LYP/6-311G(d,p) in gas and water phases colored in yellow and cyan, respectively): **hdz-CH_3_** seen from (A) above and (B) from the side; **hdz-NO_2_** seen from (C) above and (D) from the side. The optimized structures of **hdz-CH_3_** and **hdz-NO_2_** can be seen in Figure S4 in Supporting Information File 1.

Computational calculations were performed in order to confirm some of the structural and vibrational aspects of the compounds. The optimized structures obtained with B3LYP/6-311G(d,p) (both in gas and water phases) were compared to the respective experimental XRD structures (Figure 3). The RMSD values for **hdz-NO_2_** in gas (0.431 Å) and water (0.405 Å) phases were lower than the respective values for **hdz-CH_3_** (0.648 Å in the gas phase and 0.623 Å in water). However, the small RMSD values in all cases showed that the calculated structures correlated pretty well with the respective experimental ones [45–46]. The biggest differences between the theoretical structures and the experimental data corresponded to the aldehyde-derived ring, containing the –CH_3_ (or –NO_2_) substituents, an effect which is more explicit in **hdz-NO_2_**, probably due to the intrinsic disorder observed in its structure.

The comparison between theoretical and experimental selected geometric parameters can be seen in Tables S1 and S2 (Supporting Information File 1) and confirms they are in good accordance. For **hdz-CH_3_**, a maximum percentage error of 3% for bonds and 2% for angles was observed, while **hdz-NO_2_** presented values of 2% and 3% for these parameters, respectively. In turn, the intramolecular interaction OH···N1 showed maximum errors of 2% (**hdz-CH_3_**) and 0% (**hdz-NO_2_**). In general, the structures calculated involving the IEFPCM formalism (water phase) displayed the best agreement with the experimental data.

**Figure 4:**
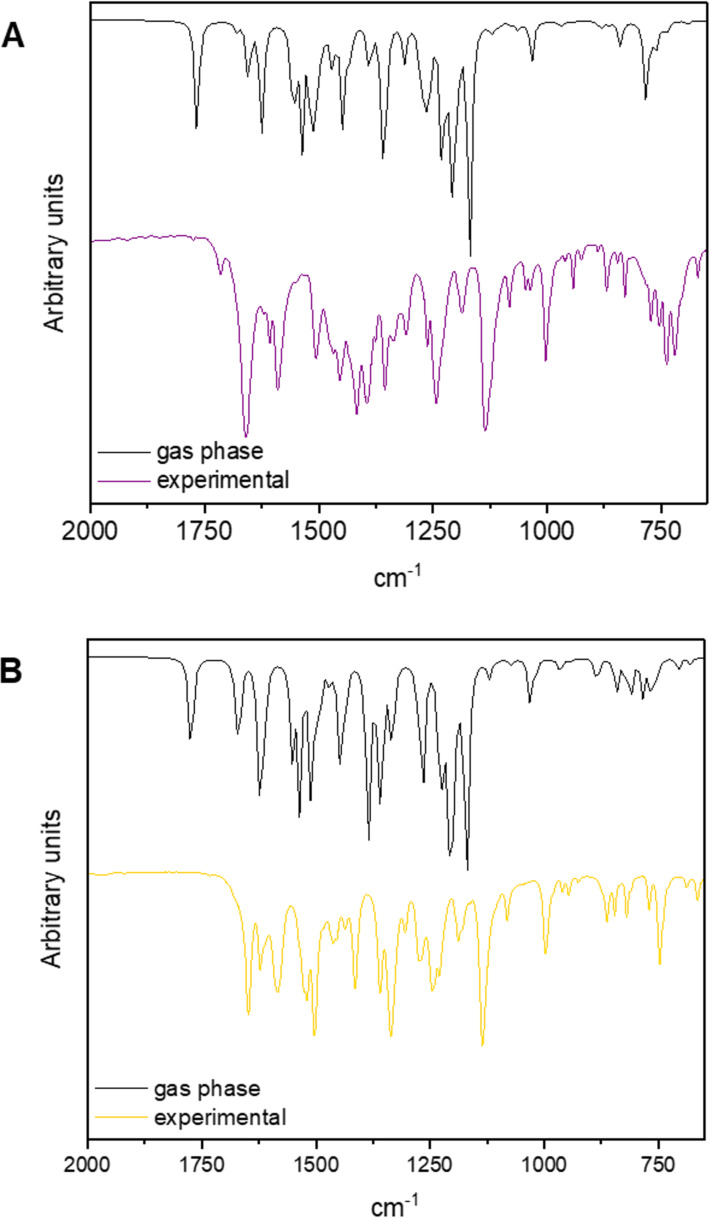
Mid-infrared spectra of the compounds. Experimental conditions: KBr pellets, room temperature. Calculated conditions: gas phase, level of theory B3LYP/6-311G(d,p). (A) Overlapping of the theoretical (black) and experimental (purple) spectra of **hdz-CH_3_**. (B) Overlapping of the theoretical (black) and experimental (yellow) spectra of **hdz-NO_2_**.

Regarding the vibrational characterization, infrared spectra were also calculated with both B3LYP/6-311G(d,p) – gas phase – and B3LYP/6-311G(d,p)/IEFPCM – water – for **hdz-CH_3_** and **hdz-NO_2_** and fitted very well with the respective experimental data, especially in the lower-frequencies region, i.e., below 2000 cm^−1^ (Figure 4 and Tables S3 and S4 in Supporting Information File 1). It is worth noting that the assignments of experimental absorptions were performed not only with the aid of DFT calculations, but also checked by comparing them to the vibrations of the respective carbonyl and hydrazide precursors.

Although the phenol-related ν(O–H) bands could not be accurately identified due to overlapping with the water stretching modes, theoretical results indicate that the frequency in **hdz-CH_3_** (3407 cm^−1^) is higher than that in **hdz-NO_2_** (3326 cm^−1^), suggesting a lower bond force constant in the latter. Differences were observed in the hydroxy bending vibrations as well, and those were perfectly observable in the spectra: δ_ip_(C–O–H) and δ_oop_(O–H) modes were assigned, respectively, at 1376 and 720 cm^−1^ for **hdz-CH_3_**, and at 1359 and 747 cm^−1^ for **hdz-NO_2_**. Interestingly, DFT showed that, while these vibrations are “clean” in **hdz-CH_3_**, they were coupled with NBA ring movements in **hdz-NO_2_**. Therefore, the IR results confirm the stronger character of the intramolecular H-bond in the nitro-substituted *N*-acylhydrazone. As previously observed for other compounds of this class, the azomethine ν(C=N) modes are barely susceptible to interactions involving the nitrogen atom, being located at 1620 cm^−1^ in **hdz-CH_3_** and at 1622 cm^−1^ in compound **hdz-NO_2_**.

Other typical bands of *N*-acylhydrazones were also attributed, such as ν(C=O) at 1658 (**hdz-CH_3_**) and 1648 cm^−1^ (**hdz-NO_2_**), as well as ν(N–N) at 1003 (**hdz-CH_3_**) and 997 cm^−1^ (**hdz-NO_2_**). The ν_as_(NO_2_) and ν_sym_(NO_2_) vibrations of the nitro group in **hdz-NO_2_** were identified as medium and strong intensity bands, respectively, at 1519 and 1336 cm^−1^. These modes were calculated at 1608 and 1379 cm^−1^ in the gas phase.

**Figure 5:**
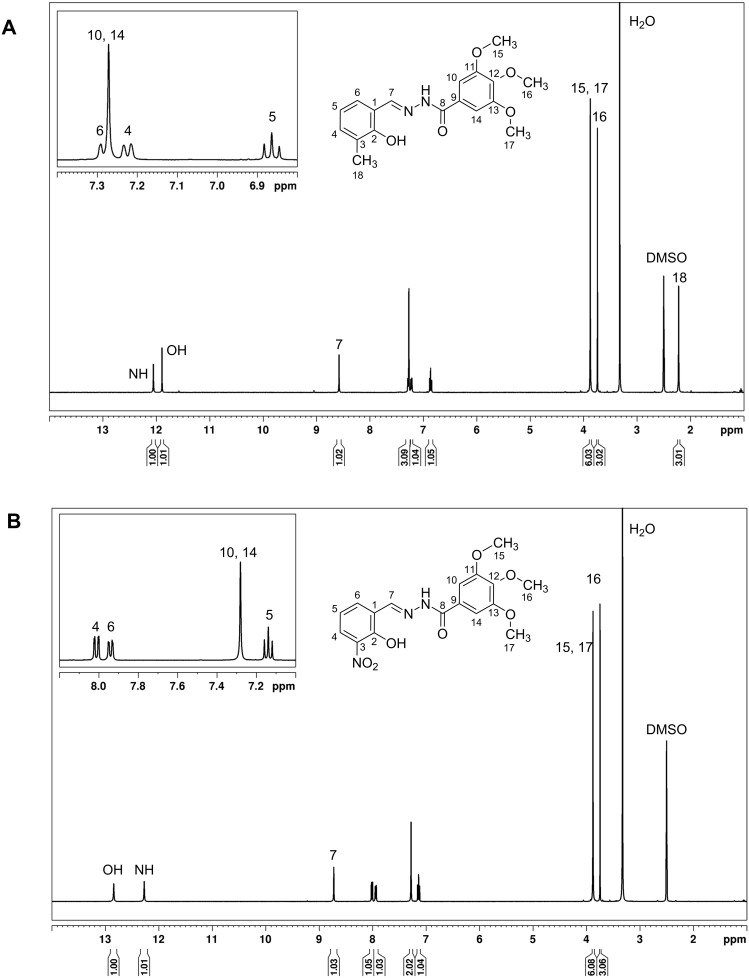
^1^H NMR (400 MHz) spectra of (A) **hdz-CH_3_** and (B) **hdz-NO_2_** in DMSO-*d*_6_ at 25 °C.

Although *N*-acylhydrazones are usually prone to undergo speciation in DMSO-*d*_6_ solution [47], ^1^H NMR measurements showed the existence of only one set of signals in the spectra of **hdz-CH_3_** and **hdz-NO_2_** (Figure 5A and 5B, respectively). ^13^C NMR and 2D homonuclear (COSY) and heteronuclear (^13^C,^1^H-HSQC and HMBC) experiments were employed for the full characterization of these hydrazones, and the spectra can be seen in Supporting Information File 1, Figures S5–S12.

Both compounds exhibit only one NH signal around 12 ppm, indicating the presence exclusively of the (*E*)-isomer in solution. Furthermore, steric hindrance in these cases allow only for the formation of the *antiperiplanar* conformation around the conjugated amide single bond. Full assignments, with the chemical shifts and coupling constants, can be found in Table S5 of Supporting Information File 1. The azomethine hydrogen H7 appears as a singlet at 8.58 ppm for **hdz-CH_3_** and is slightly deshielded in the nitro-derivative, resonating at 8.74 ppm, since the electron density-withdrawing substituent in **hdz-NO_2_** increases the electrophilic character of C7.

In both **hdz-CH_3_** and **hdz-NO_2_**, methoxy hydrogens occur as a pair of singlets: H15 and H17 at 3.88 ppm, and H16 at 3.74 ppm. Methyl H18 in **hdz-CH_3_** appears as a more shielded singlet at 2.22 ppm. Regarding the aromatic region of this compound, the doublet related to H6 (7.28 ppm) is partially overlapped with the singlet appointed to H10 and H14 (7.27 ppm). These signals were unequivocally assigned using the 2D COSY and HMBC experiments. The most shielded doublet at 7.22 ppm corresponds to H4, and H5 occurs as a triplet at 6.86 ppm. With respect to the aromatic hydrogen atoms of **hdz-NO_2_**, the singlet at 7.28 ppm was assigned to the equivalent H10 and H14. In this case, not only there was no overlapping of signals but also H4 appears more deshielded (8.01 ppm) when compared to **hdz-CH_3_**. H5, in turn, appears as a triplet at 7.14 ppm.

The presence of the electron-withdrawing NO_2_-substituent in the aldehyde-derived portion of **hdz-NO_2_** caused a strong deshielding of the hydroxy group (assigned at 12.84 ppm) due to the removal of electron density on the carbon adjacent to –OH. On the other hand, in **hdz-CH_3_**, the presence of the methyl substituent moderately shields this proton (11.89 ppm). A comparison of the H7 and –OH chemical shifts of the related hydrazones **hdz-CH_3_** and **hdz-NO_2_** indicates that, also in solution, the intramolecular H-bond is stronger in the latter.

Since *N*-acylhydrazones may be susceptible to hydrolysis, especially those containing a hydroxy group in *ortho*-position relative to the azomethine group as the intramolecular H-bond between the phenolic hydrogen and double-bonded nitrogen activates the azomethine carbon for a nucleophilic attack by a solvent molecule, an important step in the development of a new bioactive hydrazonic derivative is the assessment of its stability in aqueous medium. Thus, the electronic absorption spectra of **hdz-CH_3_** and **hdz-NO_2_** were recorded in a 10% DMSO/buffer solution (pH 7.4) immediately after preparation and at regular time intervals.

**Figure 6:**
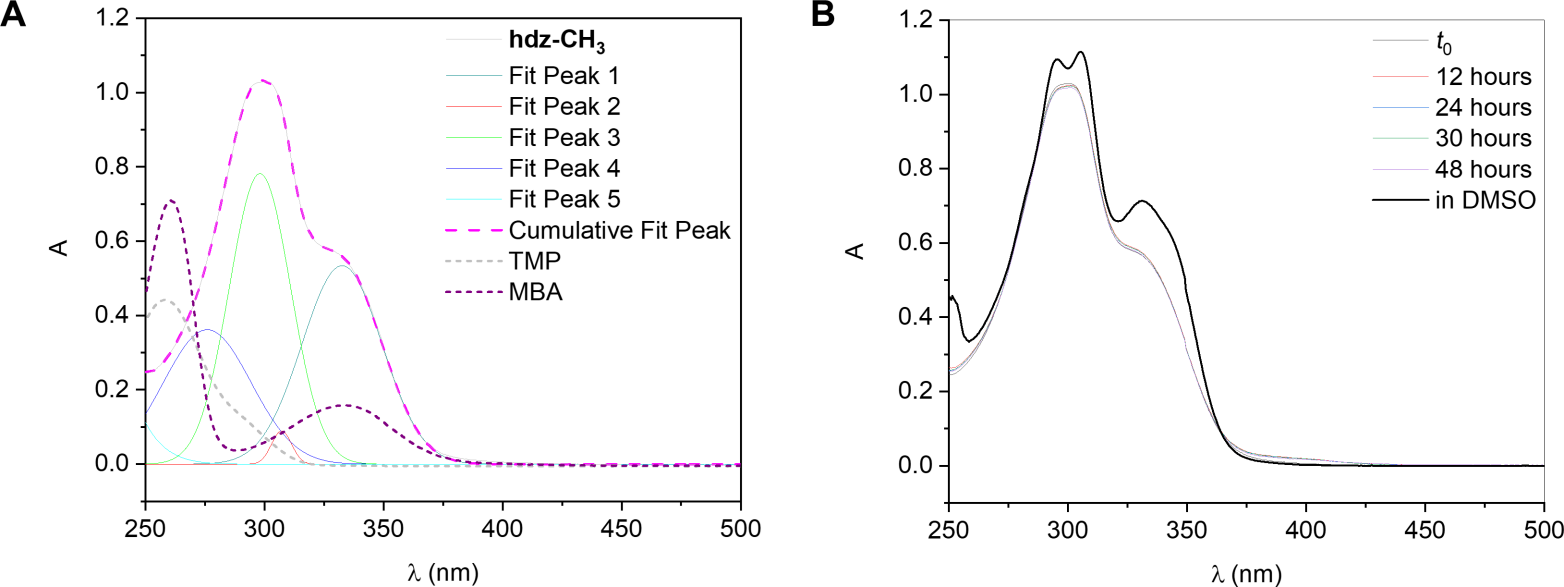
Electronic absorption spectra in a selected wavelength region for a solution of **hdz-CH_3_** in 10% DMSO/HEPES mixture (pH 7.4). Experimental conditions: *l* = 1.0 cm and *T* = (25.0 ± 0.1) °C. (A) Gaussian fitting of the bands in the spectrum at *t*_0_, along with the spectra of precursors TMP (dotted gray curve) and MBA (dotted purple). Deconvolution performed with Origin software. (B) Spectra measured at *t*_0_ and after 12, 24, 30, and 48 h. Absorption in pure DMSO (black curve) was added for the sake of comparison.

The UV–vis spectrum of **hdz-CH_3_** between 250 and 450 nm (Figure 6A) shows two multicomponent absorptions centered at 298 (ε_app_ = 20,700 ± 40 L mol^−1^ cm^−1^) and 338 nm (10,000 ± 25 L mol^−1^ cm^−1^), which could be fitted to the sum of five gaussian bands. Of these, the one at 276 nm and that of very low intensity at 307 nm were tentatively assigned by comparison with the spectra of the precursors to transitions mainly localized in the TMP ring. Nevertheless, a contribution of the phenol-containing MBA ring to the component at 276 nm cannot be ruled out. On the other hand, the constituent at 333 nm was attributed to transitions from MBA. Finally, the component at 298 nm possesses no correlates in the precursors’ absorption profiles, being consequently assigned to the hydrazone moiety, meaning that it probably involves a transition delocalized throughout the molecule (i.e., electronic density moving from one ring to the other).

No significant changes were observed in the **hdz-CH_3_** spectra during the 48 hours of follow-up study in solution (Figure 6B), indicating that the compound is stable in a water-rich medium. The spectrum of **hdz-CH_3_** in DMSO, a solvent in which *N*-acylhydrazones are usually considered long-lived species, is included in the figure for the sake of comparison. Thus, the presence of the methyl electron-donating group decreases the compound’s hydrolysis rate and therefore improves its suitability for uses demanding physiological-like conditions.

**Figure 7:**
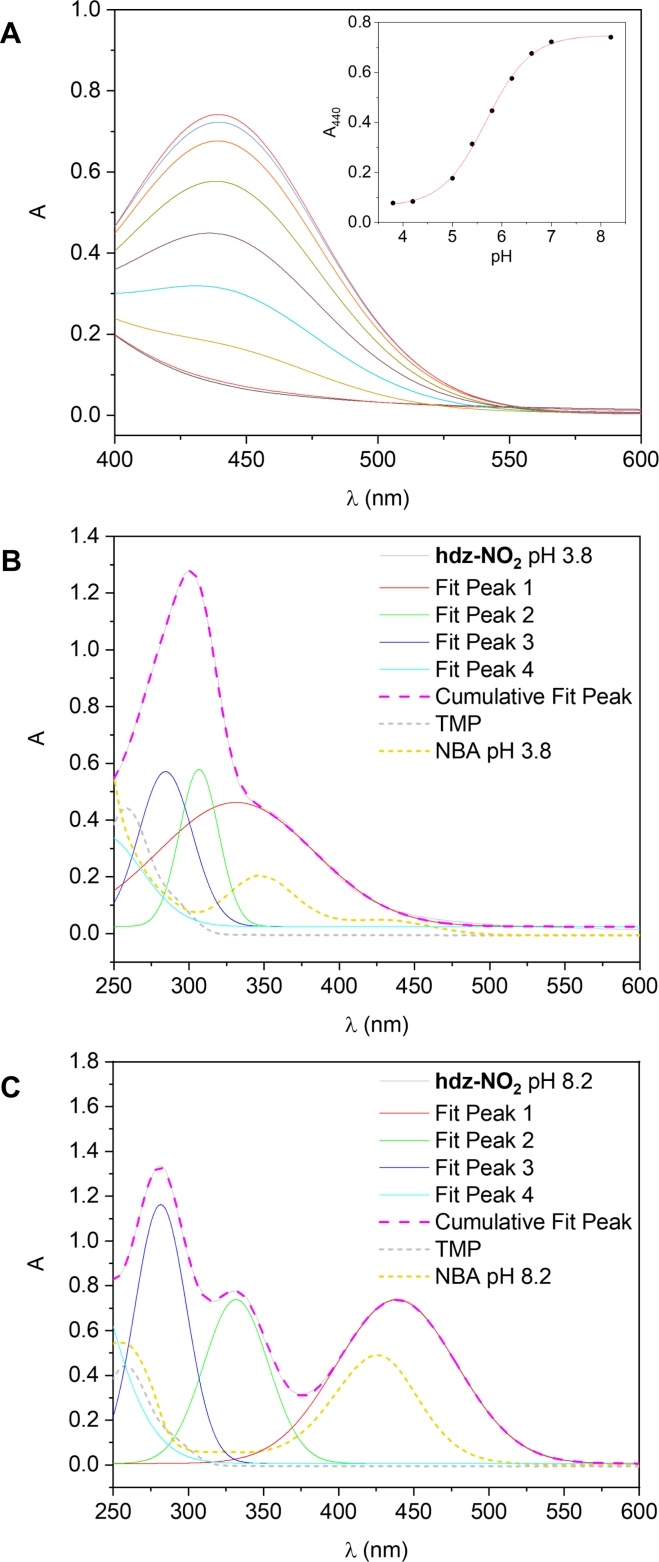
Electronic absorption spectra of **hdz-NO_2_** in selected wavelength regions. Experimental conditions: *l* = 1.0 cm and *T* = (25.0 ± 0.1) °C. (A) Deprotonation band centered at 440 nm, measured in different 10% DMSO/buffer mixtures (pH ranging from 3.8 to 8.2). Inset: A_440_ versus pH with sigmoidal fitting. Gaussian fitting of the bands in a solution of the hydrazone in (B) 10% DMSO/acetate buffer (pH 3.8) and (C) 10% DMSO/Tris-HCl buffer (pH 8.2). Deconvolution performed with Origin software. Spectra of precursors TMP (dotted gray curve) and NBA (dotted dark yellow) were included to aid in band attribution.

On the other hand, the presence of the –NO_2_ group has a very pronounced effect on the p*K*_a_ of the phenol moiety and, to a lesser extent, on the stability of the resulting hydrazone: a considerable amount of **hdz-NO_2_** deprotonates immediately upon dilution in the aqueous-rich medium at pH 7.4, affording a deep yellow solution due to phenolate-based absorptions centered at around 440 nm. For this reason, we decided to investigate this deprotonation by registering the UV–vis spectra of a series of **hdz-NO_2_** 10% DMSO/buffer (acetate, phosphate or Tris-HCl) solutions with different pH values, ranging from 3.8 to 8.2 (Figure 7A). By plotting the absorbance at λ_max_ as a function of pH and then fitting the curve with a sigmoidal function (Figure 7A, inset), an apparent p*K*_a_ of 5.68 ± 0.02 was obtained. This is quite lower than the p*K*_a_ of 2-nitrophenol (around 7.2), but a similar experiment by us demonstrated that it is higher than the one of the precursor 2-hydroxy-3-nitrobenzaldehyde (4.80 ± 0.04), since the aldehyde group has a stronger electron-withdrawing power than the hydrazone moiety.

Because of this particularity, we analyzed individually the absorption patterns of fully protonated and phenol-deprotonated **hdz-NO_2_** at the pH values of 3.8 and 8.2, respectively. When the phenol group is protonated, the spectrum of this hydrazone is very similar to that observed for **hdz-CH_3_**, with a multicomponent band and a shoulder centered, correspondingly, at 300 and 343 nm (Figure 7B). We propose that the latter is exclusively related to a broad gaussian component calculated at 331 nm, which was assigned to a protonated NBA transition through comparison with the spectrum of this precursor at pH 3.8 (dotted dark yellow curve). On the other hand, under similar arguments, the constituent at 284 nm is probably related to a TMP-based transition. In contrast, the intense component at 307 nm has no parallel in the spectra of precursors and was consequently attributed to a hydrazone-involving process, meaning, once again, that it probably involves a transition delocalized throughout the molecule. At pH 8.2, the scenario is very different: due to phenol deprotonation, the spectrum now displays three well-defined bands centered at 281, 331 and 440 nm that can be fitted by the sum of the same number of gaussian components at 282, 332 and 439 nm, which perfectly match the individual absorptions observed (Figure 7C). Comparing the contributing constituents at pH 3.8 and pH 8.2, we can conclude that the two less energetic ones are bathochromically shifted by 25 (moving from 307 to 332) and 108 (from 331 to 439) nm in the phenolate form of **hdz-NO_2_**, being the higher energy component quite unsusceptible to deprotonation and therefore confirming our assignment as a TMP-involving transition.

From the p*K*_a_ determined for the phenol group of the nitro-substituted hydrazone, it is evident that deprotonation is almost complete at pH 7.4. For this reason, the intramolecular H-bond identified both in the solid state (XRD, IR) and in solution (^1^H NMR) is probably absent under physiological or pseudo-physiological conditions.

Although still stable at pH 7.4, the **hdz-NO_2_** absorptions lose intensity by around 10% along the first 48 hours after dilution in buffer (Figure S13 in Supporting Information File 1). The mechanism associated with the hydrolysis of *N*-acylhydrazones involves the protonation of the azomethine nitrogen (in this case, N1), followed by the nucleophilic attack of a water molecule on the carbon bound to it (C7), culminating in the generation of a carbinolamine intermediate. Decomposition of this species gives the respective carbonyl compound and *N*-acylhydrazide [48–49]. In the phenol-deprotonated form of **hdz-NO_2_**, both electron-withdrawing groups (namely, nitro and azomethine) compete for the delocalized negative charge coming from the *ortho* phenolate oxygen. Even though the azomethine group has a weaker deactivating effect on the ring than the nitro group, the electron density obtained by it through this mechanism increases the basicity of the N1 atom. In fact, the proton affinity (PA) of each hydrazone was calculated and the values suggest that **hdz-NO_2_** has a higher tendency to be protonated in N1 than **hdz-CH_3_** at about 2.40 kcal mol^−1^ (PA equal to −169.711 kcal mol^−1^ for the former and −167.316 kcal mol^−1^ for the latter), favoring the hydrolysis of the nitro-containing compound.

## Conclusion

Two new structure-related 3,4,5-trimethoxybenzoyl-containing *N*-acylhydrazones, bioinspired by the hallucinogenic natural compound mescaline, were synthesized and fully characterized, both in solution and in the solid state. The compounds are derived from *meta*-substituted salicylaldehydes comprising either a methyl or a nitro group. In both cases, XRD confirmed the obtention of the (*E*)-isomer, in an *anti*-conformation.

An intramolecular H-bond involving the phenolic hydroxy group and the azomethine nitrogen N1 was identified in the solid state, and seems to be maintained in DMSO-*d*_6_ solution. It is worth noting that the presence of the electron-withdrawing nitro substituent in **hdz-NO_2_** makes the interaction stronger. An IR spectroscopy study, which was supported by computational calculations, as well as a complete NMR characterization of both compounds, align with the crystallographic observations surrounding the stronger character of this bond in the nitro-substituted hydrazone. Nevertheless, this interaction should not subsist for **hdz-NO_2_** in a more physiological environment, since the presence of an *ortho*-nitro group affects in a significant way the p*K*_a_ of the phenol: an apparent value of 5.68 ± 0.02 was obtained.

In spite of this difference in acidity, both hydrazones are stable at physiological-like conditions, especially **hdz-CH_3_**, as deprotonation of the phenol group also impacts the basicity of N1, increasing it and thus turning **hdz-NO_2_** more susceptible to hydrolysis. This is also in conformity with the calculated proton affinity values for each compound, which indicate higher propensity towards hydrolysis for the nitro-derivative (−169.711 kcal mol^−1^) over the methyl-containing one (−167.316 kcal mol^−1^) when comparing their tendency to be protonated at N1, which constitutes the first step in the mechanism.

Overall, the *N*-acylhydrazones presented in this work are pure, well-characterized from structural and spectroscopic points of view, stable at physiological pH, and contain an ONO set of donor atoms potentially able to target trivalent metal ions. For this reason, we feel quite comfortable to state that these compounds are promising, and deserve deeper studies regarding their interactions with, for example, aluminum(III) and cell toxicity assessments.

## Experimental

All solvents and reagents were purchased from commercial suppliers Sigma-Aldrich and Vetec in the highest purity available and used without further purification.

### Syntheses of the compounds

**Scheme 2:**
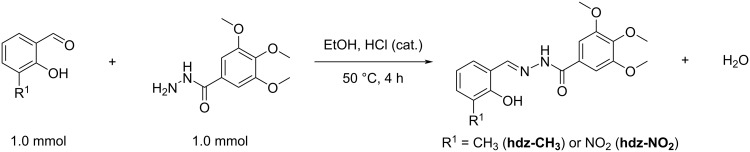
General scheme for the synthesis of the studied hydrazones.

Compounds were synthesized by modifying the existing methodology in the literature [50]. The compounds were prepared by condensation between 3,4,5-trimethoxybenzoic acid hydrazide (TMP, 1.0 mmol, 0.226 g) and 2-hydroxy-3-methylbenzaldehyde (MBA, 1.0 mmol, 0.136 g), for **hdz-CH_3_**, or 2-hydroxy-3-nitrobenzaldehyde (NBA, 1.0 mmol, 0.167 g), for **hdz-NO_2_**, in 20 mL ethanol (Scheme 2). One drop of concentrated HCl was added to the mixture as a catalyst. After stirring at 50 °C for 4 h, the mixture was cooled to room temperature and set for slow evaporation of the solvent.

3-Methylsalicylaldehyde 3,4,5-trimethoxybenzoyl hydrazone, **hdz-CH_3_**. Yield 78%; mp 180 ± 1 °C; ^1^H NMR (400 MHz, DMSO-*d*_6_) δ (ppm) 2.22 (s, 3H), 3.74 (s, 3H), 3.88 (s, 6H), 6.86 (t, ^3^*J*_HH_ = 7.52 Hz, 1H), 7.22 (d, ^3^*J*_HH_ = 7.52 Hz, 1H), 7.27 (s, 2H), 7.28 (d, ^3^*J*_HH_ = 7.52 Hz, 1H), 8.58 (s, 1H), 11.89 (s, 1H), 12.05 (s, 1H); MS (*m*/*z*) 344.18 (calcd. 344.36).

3-Nitrosalicylaldehyde 3,4,5-trimethoxybenzoyl hydrazone, **hdz-NO_2_**. Yield 44%; mp 205 ± 1 °C; ^1^H NMR (400 MHz, DMSO-*d*_6_) δ (ppm) 3.74 (s, 3H), 3.88 (s, 6H), 7.14 (t, ^3^*J*_HH_ = 7.92 Hz, 1H), 7.28 (s, 2H), 7.94 (dd, ^3^*J*_HH_ = 7.92 Hz, ^4^*J*_HH_ = 1.49 Hz, 1H), 8.01 (dd, ^3^*J*_HH_ = 7.92 Hz, ^4^*J*_HH_ = 1.49 Hz, 1H), 8.74 (s, 1H), 12.27 (s, 1H) 12.84 (s, 1H); MS (*m*/*z*): 375.44 (calcd. 375.33).

### Chemical characterization

A Perkin-Elmer 100 FT-IR spectrometer was used to record the mid-infrared spectra of the solid samples in spectroscopic grade potassium bromide (KBr). Thermogravimetric analyses were performed using a Pyris 1 TGA thermoanalyzer (Perkin-Elmer), at 10 °C min^−1^ heating rate, under nitrogen flow (20 mL min^−1^), from 25 to 350 °C. Melting points were determined in triplicate using a Fisatom Model 431 apparatus. Hydrogen and carbon nuclear magnetic resonance spectra (NMR), homonuclear ^1^H,^1^H (COSY and NOESY) and heteronuclear ^1^H,^13^C (HSQC, HMBC) experiments were recorded on a 400 MHz Avance III (Bruker, Billerica, MA) spectrometer. Samples were dissolved in 0.5 mL DMSO-*d*_6_ and spectra were referenced based on the residual solvent signal (quintet at 2.50 ppm for ^1^H and septet at 39.52 for ^13^C). Mass spectra were obtained on a Trace 1300 gas chromatograph connected to ISQ QD single quadrupole mass spectrometer (Thermo Fisher Scientific Inc., Waltham, MA, USA). Samples were prepared in dichloromethane at 1 mg mL^−1^ concentration.

#### Molecular absorption spectroscopy

Molecular absorption spectra were recorded on Agilent Cary 100 conc UV–visible spectrophotometer between the range of 200 and 800 nm in quartz cuvettes. Stock solutions of the compounds were prepared in spectroscopic grade DMSO at 5 × 10^−3^ mol L^−1^. Dilutions (5 × 10^−5^ mol L^−1^) were prepared in DMSO/buffer (50 × 10^−3^ mol L^−1^) in a 10:90 (v/v) ratio and kept at 25 °C during the whole experiment. Spectra were recorded immediately after preparation of the solutions and at defined time intervals. The changes in absorbance intensity were used to calculate the percentage decrease in concentration of the compound with respect to the first reading and data were processed using the OriginPro 21 software.

#### X-ray diffraction

Single crystals of **hdz-CH_3_** and **hdz-NO_2_** suitable for X-ray diffraction were obtained from the slow evaporation of the syntheses’ mother liquors. They were analyzed in a D8-Venture Bruker diffractometer equipped with Mo Κα X-ray source at 293 K. Diffraction images were collected with a Photon III area detector and the frames were integrated with the Bruker SAINT software using a narrow-frame algorithm [51]. Absorption correction was conducted with the multi-scan method in SADABAS software (APEX3 system) [52]. The structures were solved with directed methods in ShelxS [53] and refined with full-matrix least-square in ShelxL [53], implemented in WinGX [54] and ShelxLE [55] platforms. Non-hydrogen atoms were located from the electron density maps and anisotropically refined. C–H hydrogens were ride over the parent carbon with H(Uiso) = 1.2 or 1.5 C (Ueq). N–H and O–H hydrogens were located from the difference maps and isotropically refined using adequate restrains. Disordered nitro group oxygen in **hdz-NO_2_** was treated as a two-position model (O6A, O6B), being O6A the major position with 72% of occupancy. Figures were prepared with Mercury [56], Fourier maps, data and tables were prepared with Platon [57], and the Hirshfeld surfaces and fingerprint plots were calculated from the CIF files using Crystal Explorer [58]. Potential electrostatic maps and electron density surfaces were calculated with DFT method from the CIF files using TONTO, also implemented in Crystal Explorer [58].

#### Computational methods

All calculations were done with the Gaussian 16 package [59] using the B3LYP exchange and correlation functional [60–61]. The 6-311G(d,p) basis set, which included polarization functions, was employed for all atoms [62–63]. The calculations were performed in the gas and water phases. This last was used the implicit solvation effect with the IEFPCM formalism [64]. The absence of imaginary frequencies showed that the obtained structures corresponded to energy minimums. The proton affinity (PA = E_HL_^+^ − E_L_) was calculated from the energy difference between each protonated molecule and the respective neutral molecule [65]. The Root Medium Square Difference (RMSD) between each experimental and theoretical structure was calculated with the Pymol tool [66].

## Supporting Information

Supporting Information contains Fourier difference maps (Figure S1), Hirshfeld surface analyses (Figures S2 and S3), optimized calculated structures (Figure S4), experimental and calculated geometric parameters (Tables S1 and S2) and vibrational assignments (Tables S3 and S4), ^13^C, COSY, HMBC and HSQC spectra (Figures S5–S12) and the full NMR characterization (Table S5) for both hydrazones reported herein. Hydrolytic stability of **hdz-NO_2_** at pH 7.4 is also shown (Figure S13). Accession codes CCDC 2254324 and 2255022 contain the supplementary crystallographic data for the new hydrazones **hdz-CH_3_** and **hdz-NO_2_**, respectively. These data can be obtained free of charge via https://www.ccdc.cam.ac.uk/data_request/cif, or by emailing data_request@ccdc.cam.ac.uk, or by contacting Cambridge Crystallographic Data Centre, 12 Union Road, Cambridge CB2 1EZ, UK; fax: +44 1223 336033.

File 1 Additional information.
