# Supplementary material for: Effects of the aldehyde-derived ring substituent on the properties of two new bioinspired trimethoxybenzoylhydrazones: methyl vs nitro groups
Source: Beilstein J Org Chem. 2023 Nov 10;19:1713–27. doi: 10.3762/bjoc.19.125 (PMC10644011; doi:10.3762/bjoc.19.125)
Supplement: File 1 — Additional information. [file Beilstein_J_Org_Chem-19-1713-s001.pdf]

BEILSTEIN JOURNAL OF ORGANIC CHEMISTRY

Supporting Information

for

Effects of the aldehyde-derived ring substituent on the properties of two new bioinspired trimethoxybenzoylhydrazones: methyl vs nitro groups

Dayanne Martins, Roberta Lamosa, Talis Uelisson da Silva, Carolina B. P. Ligiero, Sérgio de Paula Machado, Daphne S. Cukierman and Nicolás A. Rey

Beilstein J. Org. Chem. **2023,** *19,* 1713–1727. doi:10.3762/bjoc.19.125

Additional information

License and Terms: This is a supporting information file under the terms of the Creative Commons Attribution License (https://creativecommons.org/licenses/by/4.0). Please note that the reuse, redistribution and reproduction in particular requires that the author(s) and source are credited and that individual graphics may be subject to special legal provisions.
The license is subject to the *Beilstein Journal of Organic Chemistry* terms and conditions: (https://www.beilstein-journals.org/bjoc/terms)

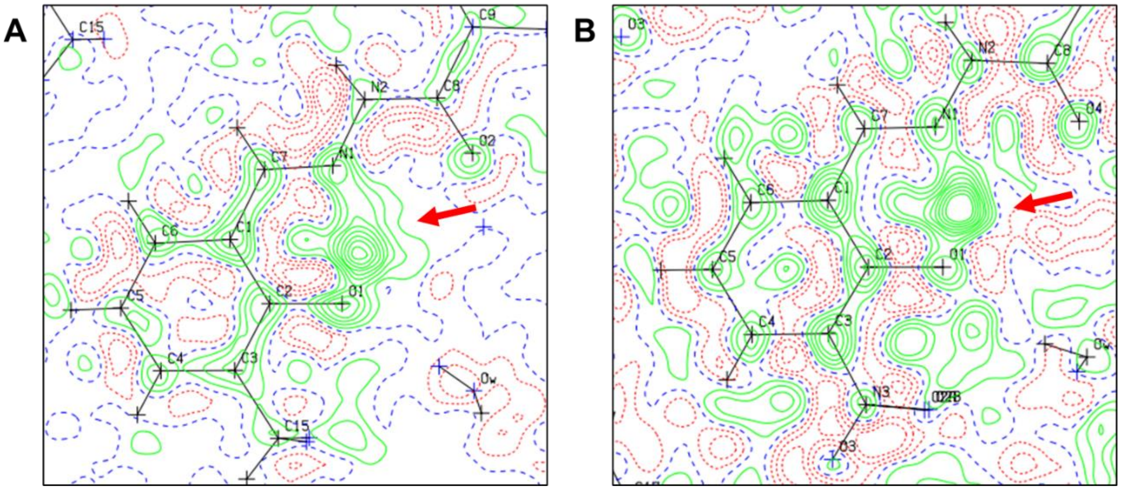

Figure S1: Fourier difference maps without the H. (**A**) **hdz-CH$_3$**; plane 16.0517*x*, −1.4375*y*, −14.0518*z*, contour level (e/Å^3) −0.25, 0.45, 0.05, resolution 26.4°. (**B**) **hdz-NO$_2$**; plane 6.3871*x*, 0.3473*y*, 8.5161*z*, contour level (e/Å^3) −0.20, 0.45, 0.05, resolution 25.3°.

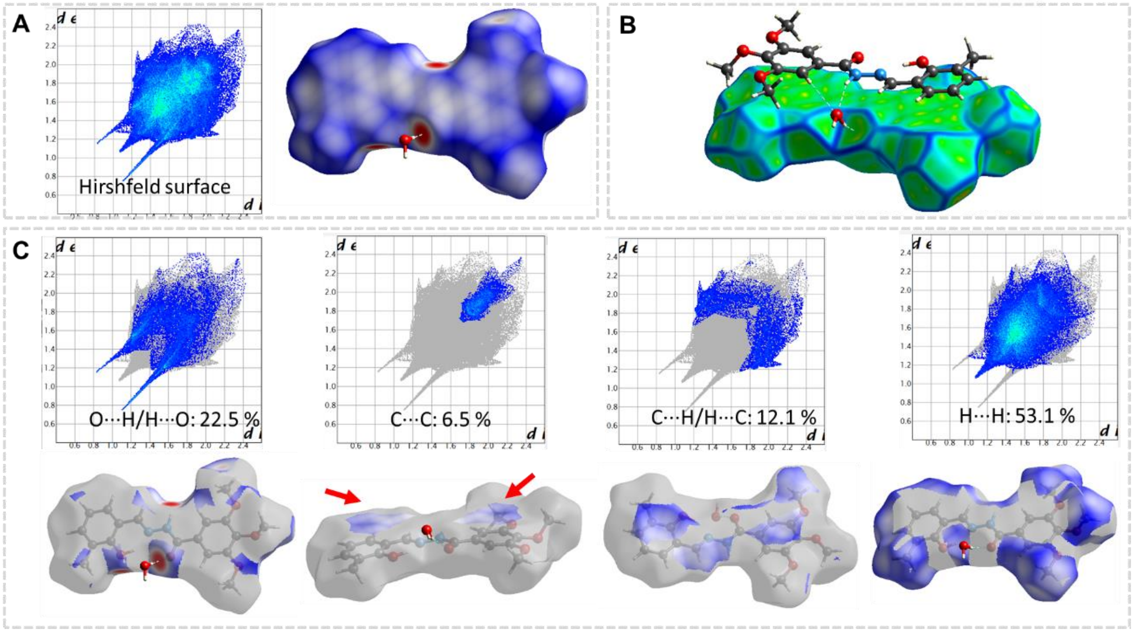

Figure S2: Hirshfeld surface for **hdz-CH$_3$**. (**A**) Hirshfeld surface and d_{norm} map (−0.5725 to 1.1747) for all interactions. (**B**) Curvedness. (**C**) d_{norm} map filtered by type of interaction and Fingerprint plots.

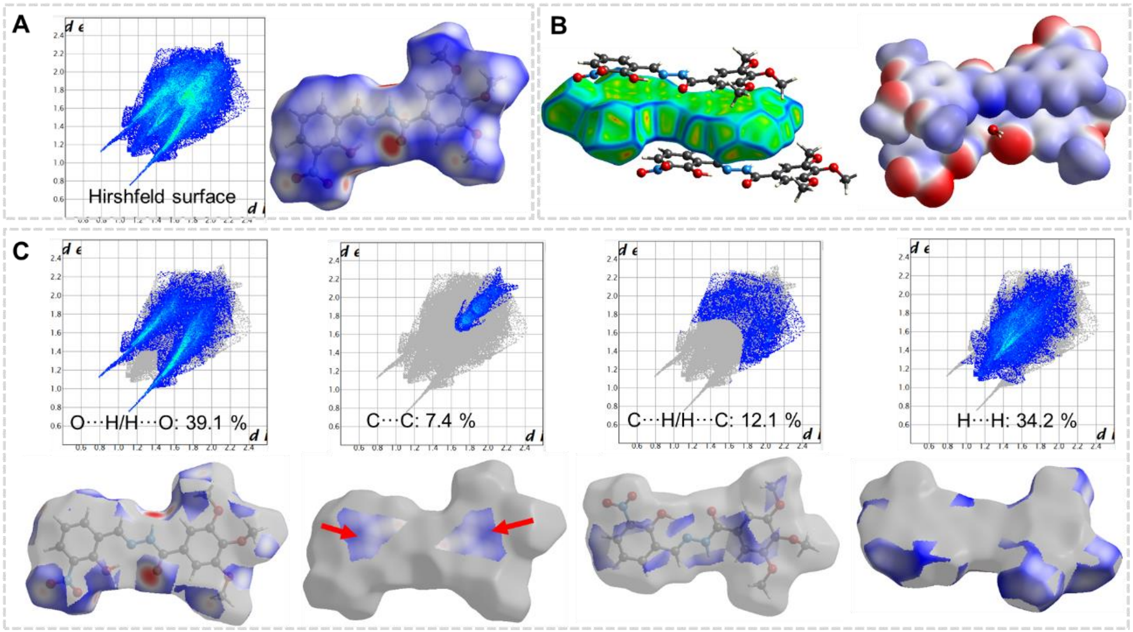

Figure S3: Hirshfeld surface for **hdz-NO$_2$**. (**A**) Hirshfeld surface and d_{norm} map (−0.5725 to 1.1747) for all interactions. (**B**) Left: curvedness. Right: electrostatic potential (−0.0811 to 0.1788 au) over the electron density surface [*isosurface* 0.008 e au^{-3}/DFT/B3LYP/6-311(d,p)]. (**C**) d_{norm} map filtered by type of interaction and Fingerprint plots.

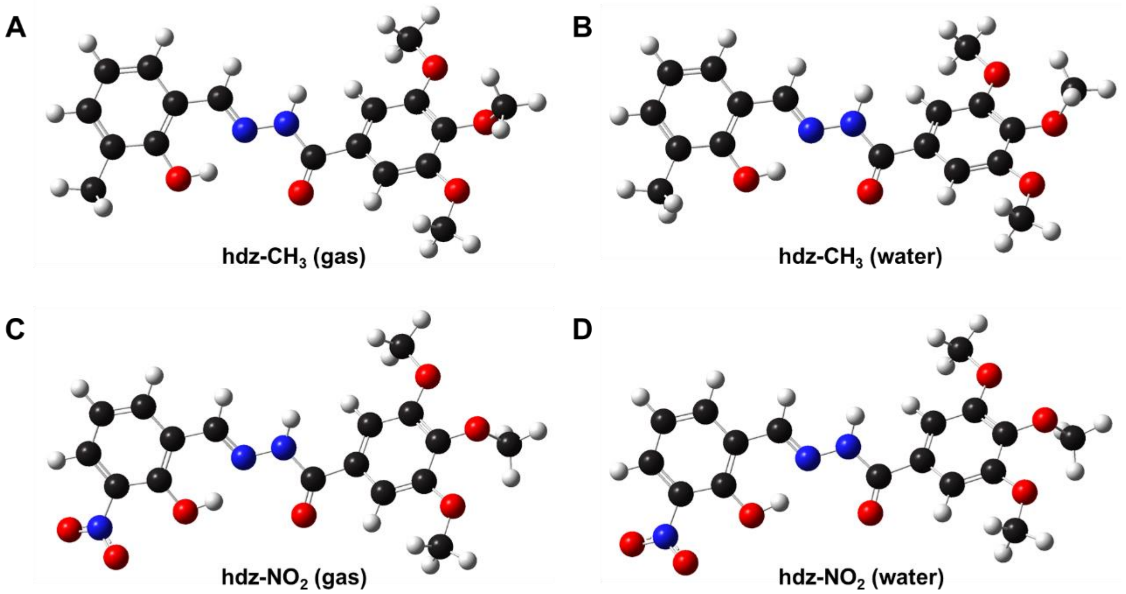

Figure S4: Optimized structures for **hdz-CH$_3$** (**A**) in the gas phase and (**B**) in water; and for **hdz-NO$_2$** (**C**) in the gas phase and (**D**) in water. Levels of theory were [B3LYP/6-311G(d,p)] and [B3LYP/6-311G(d,p)IEFPCM] for each phase (gas and water-continuum model), respectively.

Table S1: Selected experimental and theoretical [levels of theory B3LYP/6-311G(d,p) and B3LYP/6-311G(d,p)IEFPCM for gas and water phases, respectively] geometric parameters for **hdz-CH_3**.

Bond lengths (Å)	Experimental	Calculated	
		Gas phase	Water phase
C1–C2	1.405(2)	1.418	1.418
C1–C6	1.398(3)	1.407	1.407
C2–O1	1.357(2)	1.345	1.351
C3–C18	1.507(3)	1.505	1.506
C1–C7	1.450(3)	1.451	1.453
C7=N1	1.279(3)	1.286	1.287
N1–N2	1.375(2)	1.356	1.359
N2–C8	1.355(3)	1.390	1.378
C8=O2	1.226(2)	1.214	1.223
C8–C9	1.491(2)	1.499	1.498
C9–C14	1.387(3)	1.399	1.399
C9–C10	1.385(2)	1.394	1.396
C13–O5	1.368(2)	1.360	1.358
C12–O4	1.372(2)	1.364	1.369
C11–O3	1.363(2)	1.359	1.358
Bond angles (°)			
C6–C1–C2	118.9(2)	119.042	118.976
C2–C3–C18	119.7(2)	119.085	119.495
C1–C2–O1	121.8(2)	122.718	122.031
C7–N1–N2	118.9(2)	119.224	118.955
N1–N2–C8	116.9(2)	119.898	119.946
N2–C8–O2	121.0(2)	122.275	122.153
O2–C8–C9	121.4(2)	123.247	122.754
C8–C9–C10	124.2(2)	123.052	122.635
C14–C13–O5	124.2(2)	124.546	124.563
C13–C12–O4	119.4(2)	119.288	120.405
C12–C11–O3	115.3(2)	115.310	115.485

Table S2: Selected experimental and theoretical [levels of theory B3LYP/6-311G(d,p) and B3LYP/6-311G(d,p)IEFPCM for gas and water phases, respectively] geometric parameters for **hdz-NO_2**.

	Experimental	Gas	Water
Bond lengths (Å)			
C1–C2	1.411(3)	1.427	1.427
C1–C6	1.390(4)	1.400	1.400
C2–O1	1.344(3)	1.326	1.326
C3–N3	1.452(5)	1.476	1.476
C1–C7	1.456(4)	1.454	1.454
C7=N1	1.279(3)	1.284	1.284
N1–N2	1.373(3)	1.351	1.351
N2–C8	1.365(3)	1.397	1.397
C8=O2	1.219(3)	1.212	1.212
C8–C9	1.488(3)	1.495	1.495
C9–C14	1.390(3)	1.400	1.400
C9–C10	1.384(3)	1.395	1.395
C13–O5	1.370(3)	1.359	1.359
C12–O4	1.373(3)	1.362	1.362
C11–O3	1.358(3)	1.358	1.358
Bond angles (°)			
C6–C1–C2	119.7(2)	119.867	119.867
C2–C3–N3	121.1(3)	121.171	121.171
C1–C2–O1	121.6(3)	122.499	122.499
C7–N1–N2	120.0(2)	119.984	119.984
N1–N2–C8	116.5(2)	119.492	119.492
N2–C8–O2	120.5(2)	121.836	121.836
O2–C8–C9	122.2(2)	123.699	123.699
C8–C9–C10	123.6(2)	122.938	122.938
C14–C13–O5	123.8(2)	124.574	124.574
C13–C12–O4	119.6(2)	119.233	119.233
C12–C11–O3	115.3(2)	115.325	115.325

Table S3: Experimental and calculated [gas, level of theory B3LYP/6-311++G(2d,p) and water phase, B3LYP/6-311G(d,p)/IEFPCM] IR data for the main absorptions of **hdz-CH_3**, with proposed assignments.

Frequencies (cm^{-1})			Vibrational assignments
Experimental (KBr)	Calculated		
	Gas phase	Water phase	
3431 (w)	3407	3313	$\nu(O–H)_{phenol} + \nu(O–H)_{water}$
3282 (w)	3227	3223	$\nu(C–H)_{TMP\ ring}$
3175 (w)	3526	3538	$\nu(N–H)$
3063 (m)	3162	3166	$\nu_{as}(C–H)_{MBA\ ring}$
3037 (m)	3029	3072	$\nu(C–H)_{azomethine}$
2983 (m)	3138	3146	$\nu_{as}(C–H)_{methoxyl}$
2971 (m)	3129	3132	$\nu_{as}(C–H)_{methoxyl}$
2950 (m)	3109	3111	$\nu_{as}(C–H)_{methyl}$
2933 (m)	3083	3087	$\nu_{as}(C–H)_{methoxyl}$
2917 (m)	3079	3073	$\nu_{as}(C–H)_{methyl}$
2871 (m)	3030	3027	$\nu_{sym}(C–H)_{methyl}$
2836 (m)	3008	3020	$\nu_{sym}(C–H)_{methoxyl}$
1658 (s)	1762	1712	$\nu(C=O) + \delta(N–N–H)_{hydrazone}$
1620 (m)	1675	1669	$\nu(C=N)_{hydrazone} + \delta_{ip}(N=C–H)_{azomethine} + \nu(C=C)_{MBA\ ring}$
1607 (m)	1649	1645	$\nu(C=C)_{MBA\ ring}$
1590 (s)	1619	1617	$\nu(C=C)_{TMP\ ring}$
1551 (w)	1551	1561	$\delta(N–N–H)_{hydrazone}$
1505 (m)	1532	1528	$\nu(C=C)_{TMP\ ring} + \delta_{ip}(C=C–H)_{TMP\ ring}$
1469 (m)	1513	1513	$\nu(C=C)_{MBA\ ring} + \delta_{ip}(C=C–H)_{MBA\ ring}$
1454 (m)	1504	1494	$\delta(H–C–H)_{methyl} + \delta(H–C–H)_{methoxyl} + \delta_{ip}(C=C–H)_{MBA\ ring}$
1435 (sh)	1487	1485	$\delta(H–C–H)_{methoxyl} + \delta(O–C–H)_{methoxyl}$
1417 (s)	1442	1437	$\delta(O–C–H)_{methoxyl} + \delta_{ip}(C=C–H)_{TMP\ ring}$
1395 (s)	1465	1456	$\delta(H–C–H)_{methyl} + \delta(O–C–H)_{methoxyl} + \delta_{ip}(C=C–H)_{MBA\ ring}$
1376 (m)	1430	1441	$\delta_{ip}(C–O–H)_{phenol}$
1355 (s)	1383	1385	$\delta_{ip}(N=C–H)_{azomethine} + \delta(N–N–H)_{hydrazone}$
1337 (m)	1355	1348	$\nu(C–OCH_3) + \nu(C=C)_{TMP\ ring} + \delta(O–C–H)_{methoxyl} + \delta(N–N–H)_{hydrazone}$
1309 (m)	1350	1343	$\nu(C=C)_{MBA\ ring} + \delta_{ip}(C–O–H)_{phenol}$
1262 (m)	1307	1292	$\nu(C=C)_{MBA\ ring} + \nu(C–O)_{phenol}$
1243 (s)	1265	1252	$\delta_{ip}(C=C–H)_{MBA\ ring} + \delta_{ip}(C=C–H)_{TMP\ ring} + \delta_{ip}(C–O–H)_{phenol}$
1230 (sh)	1347	1348	$\nu(C=C)_{TMP\ ring}$
1135 (s)	1212	1203	$\delta(O–C–H)_{methoxyl} + \delta(N–N–H)_{hydrazone}$
1083 (m)	1189	1185	$\delta_{ip}(C=C–H)_{MBA\ ring}$
1003 (m)	1164	1161	$\nu(N–N)_{hydrazone}$
943 (w)	1116	1118	$\delta_{ip}(C=C–H)_{MBA\ ring} + \delta_{ip}(C=C–H)_{TMP\ ring}$
869 (w)	1071	1047	$\nu(O–CH_3)_{methoxyl}$
845 (w)	1062	1059	$\delta(C–C–H)_{methyl}$
829 (w)	1056	-	$\delta_{ip}(N=C–H)_{azomethine}$
773 (m)	1027	1009	TMP breathing mode + $\nu(O–CH_3)_{methoxyl}$
754 (m)	966	974	$\delta_{oop}(C–H)_{azomethine} + \delta_{oop}(C–H)_{MBA\ ring}$
737 (m)	857	855	$\delta_{oop}(C–H)_{TMP\ ring} + \delta(C=C–C)_{MBA\ ring}$
720 (m)	786	768	$\delta_{oop}(O–H)_{phenol}$
670 (w)	779	753	$\delta_{oop}(C–H)_{MBA\ ring}$

Intensities: w = weak; m = medium; s = strong; sh = shoulder.

Table S4: Experimental and calculated [gas, level of theory B3LYP/6-311++G(2d,p) and water phase, B3LYP/6-311G(d,p)/IEFPCM] IR data for the main absorptions of **hdz-NO_2**, with proposed assignments.

Frequencies (cm^{-1})			Vibrational assignments
Experimental (KBr)	Calculated		
	Gas phase	Water phase	
3455 (w)	3326	3154	ν(O–H)$_{phenol}$ + ν(O–H)$_{water}$
3222 (w)	3527	3542	ν(N–H)
3188 (sh)	3226	3227	ν(C–H)$_{TMP\ ring}$
3088 (w)	3219	3206	ν(C–H)$_{NBA\ ring}$
3008 (w)	3041	3085	ν(C–H)$_{azomethine}$
2976 (w)	3139	3146	ν_{as}(C–H)$_{methoxyl}$
2949 (w)	3088	3133	ν_{as}(C–H)$_{methoxyl}$
2936 (w)	3066	3086	ν_{as}(C–H)$_{methoxyl}$
2837 (w)	3010	3021	ν_{sym}(C–H)$_{methoxyl}$
1648 (s)	1768	1718	ν(C=O) + δ(N–N–H)$_{hydrazone}$
1622 (m)	1677	1672	ν(C=N)$_{hydrazone}$ + δ_{ip}(N=C–H)$_{azomethine}$
1615 (sh)	1664	1655	ν(C=C)$_{NBA\ ring}$
1584 (m)	1620	1615	ν(C=C)$_{TMP\ ring}$ + δ_{ip}(C=C–H)$_{TMP\ ring}$
1523 (sh)	1613	1612	ν(C=C)$_{NBA\ ring}$ + δ_{ip}(C–O–H)$_{phenol}$ + $_{ip}$(C=C–H)$_{NBA\ ring}$
1519 (m)	1608	1572	ν_{as}(NO_2) + ν(C=C)$_{NBA\ ring}$ + δ_{ip}(C=C–H)$_{NBA\ ring}$
1503 (s)	1548	1554	δ(N–N–H)$_{hydrazone}$ + ν(C=C)$_{TMP\ ring}$
1462 (m)	1504	1497	δ(H–C–H)$_{methoxyl}$
1455 (m)	1493	1495	δ(H–C–H)$_{methoxyl}$
1436 (w)	1487	-	δ_{ip}(C=C–H)$_{NBA\ ring}$ + δ(H–C–H)$_{methoxyl}$ + δ(O–C–H)$_{methoxyl}$
1414 (m)	1465	1461	δ(O–C–H)$_{methoxyl}$
1359 (m)	1432	1447	δ_{ip}(C–O–H)$_{phenol}$ + δ_{ip}(C=C–H)$_{NBA\ ring}$
1336 (s)	1379	1369	ν_{sym}(NO_2) + δ_{ip}(C–O–H)$_{phenol}$ + δ_{ip}(C=C–H)$_{NBA\ ring}$
1305 (w)	1332	1332	ν(C=C)$_{NBA\ ring}$
1272 (m)	1326	1303	ν(C–O)$_{phenol}$ + δ_{ip}(C=C–H)$_{NBA\ ring}$
1245 (m)	1274	1276	ν(C–OCH_3) + δ_{ip}(C=C–H)$_{TMP\ ring}$
1230 (m)	1261	1250	ν(C–OCH_3) + δ_{ip}(C=C–H)$_{TMP\ ring}$
1188 (m)	1259	1263	δ_{ip}(C=C–H)$_{TMP\ ring}$ + δ_{ip}(C=C–H)$_{NBA\ ring}$ + δ_{ip}(N=C–H)$_{azomethine}$
1135 (s)	1222	1225	δ(O–C–H)$_{methoxyl}$ + δ(N–N–H)$_{hydrazone}$
1081 (w)	1206	1205	δ_{ip}(C=C–H)$_{NBA\ ring}$
997 (m)	1169	1167	ν(N–N)$_{hydrazone}$ + δ(O–C–H)$_{methoxyl}$ + δ_{ip}(C=C–H)$_{aromatic}$
862 (w)	1115	1117	δ_{ip}(C=C–H)$_{aromatic}$
845 (w)	1067	1148	ν(O–CH_3)$_{methoxyl}$
819 (w)	958	961	ν(C–NO_2) + δ(C=C–C)$_{aromatic}$
770 (w)	879	863	δ(C=C–C)$_{NBA\ ring}$ + δ_{oop}(C–H)$_{TMP\ ring}$
747 (m)	818	832	NBA breathing mode + δ_{oop}(O–H)$_{phenol}$
665 (w)	757	757	δ_{oop}(C–H)$_{NBA\ ring}$

Intensities: w = weak; m = medium; s = strong; sh = shoulder.

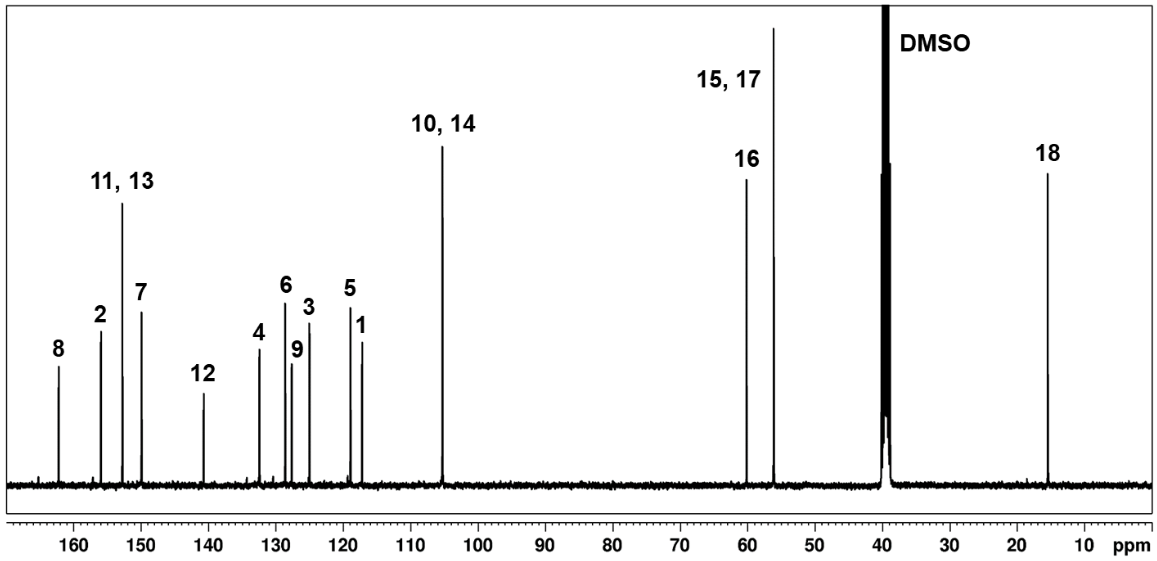

Figure S5: ^{13}C NMR (100 MHz) spectra of **hdz-CH$_3$** in DMSO-d_6 at 25 °C.

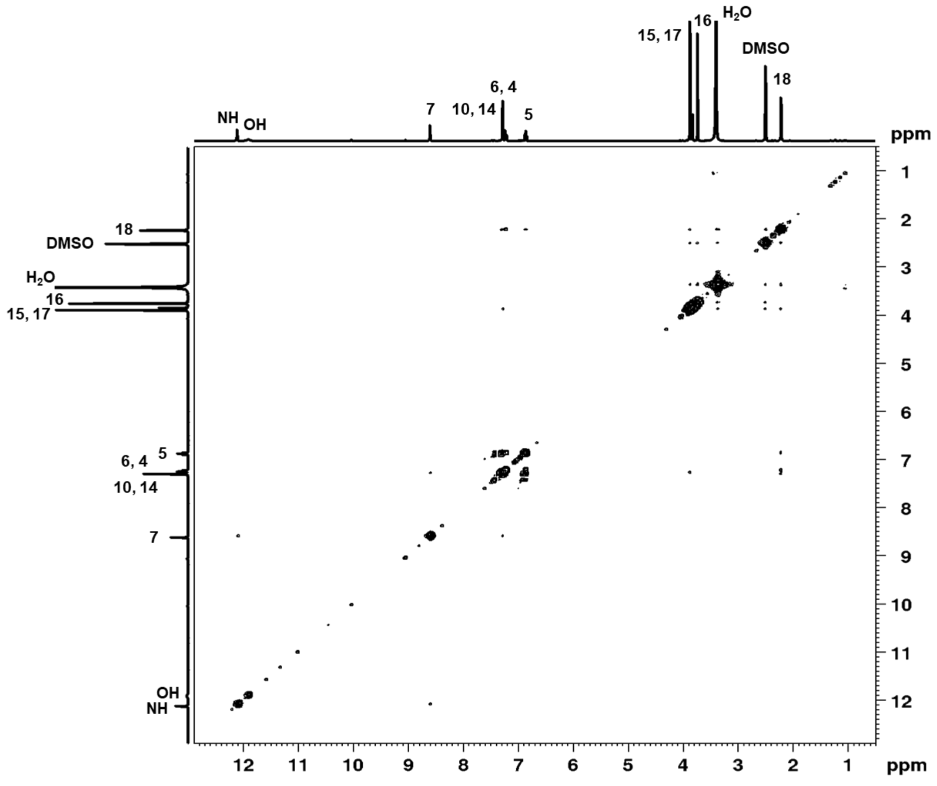

Figure S6: COSY contour map of **hdz-CH$_3$** in DMSO-d_6 at 25 °C.

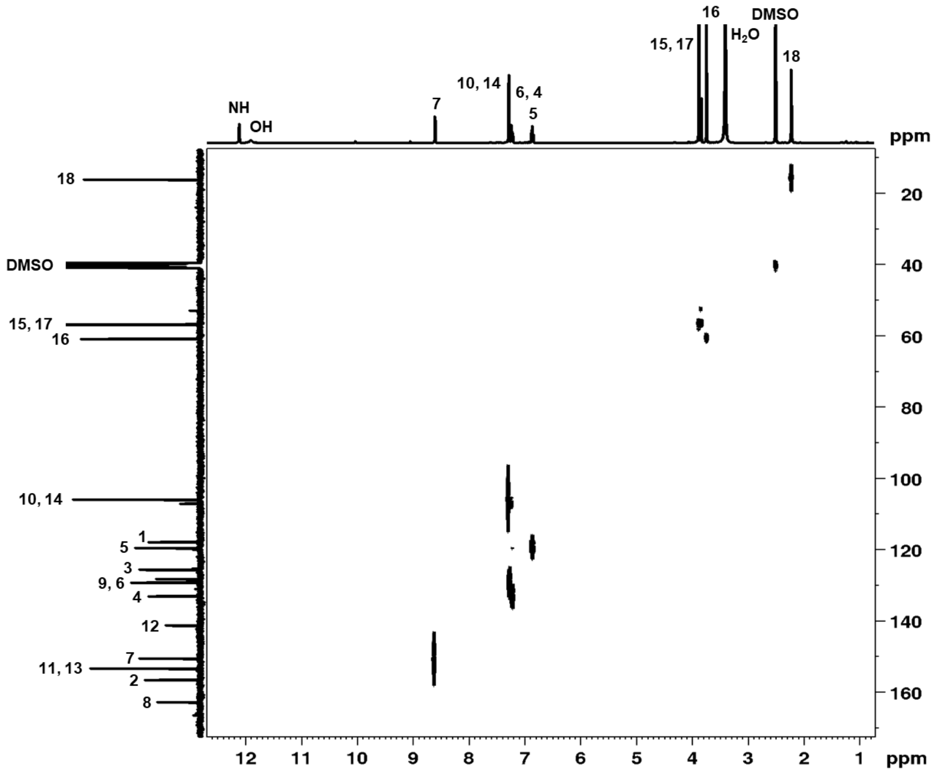

Figure S7: HSQC contour map of **hdz-CH$_3$** in DMSO-d_6 at 25 °C.

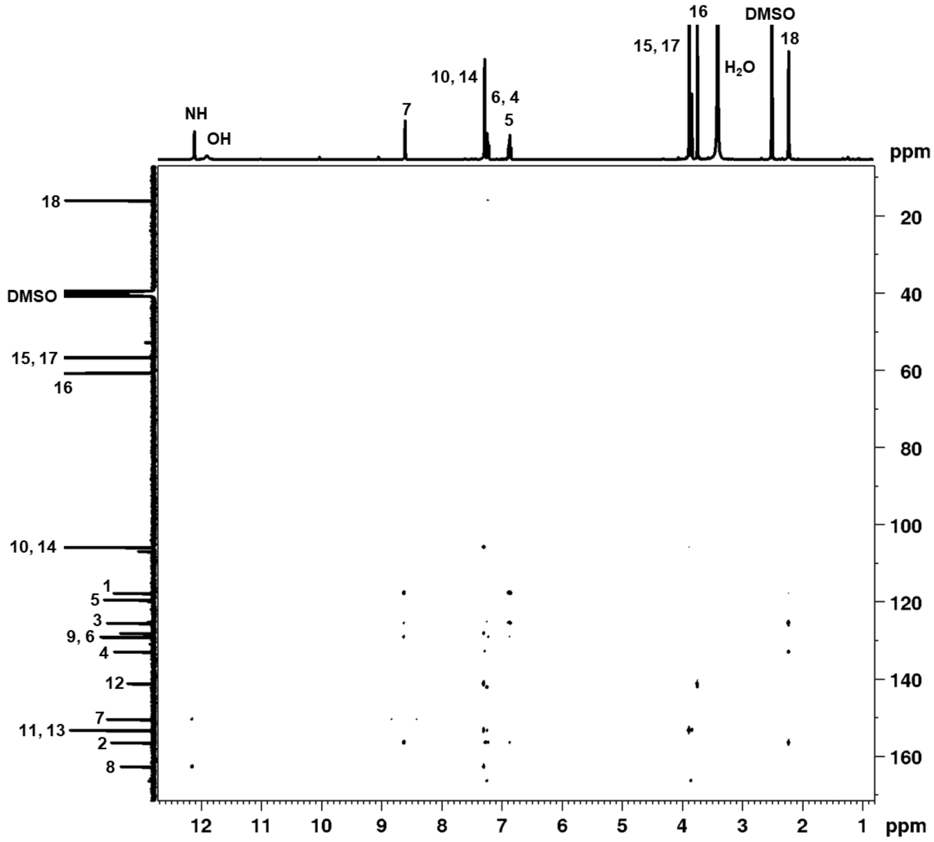

Figure S8: HMBC contour map of **hdz-CH$_3$** in DMSO-d_6 at 25 °C.

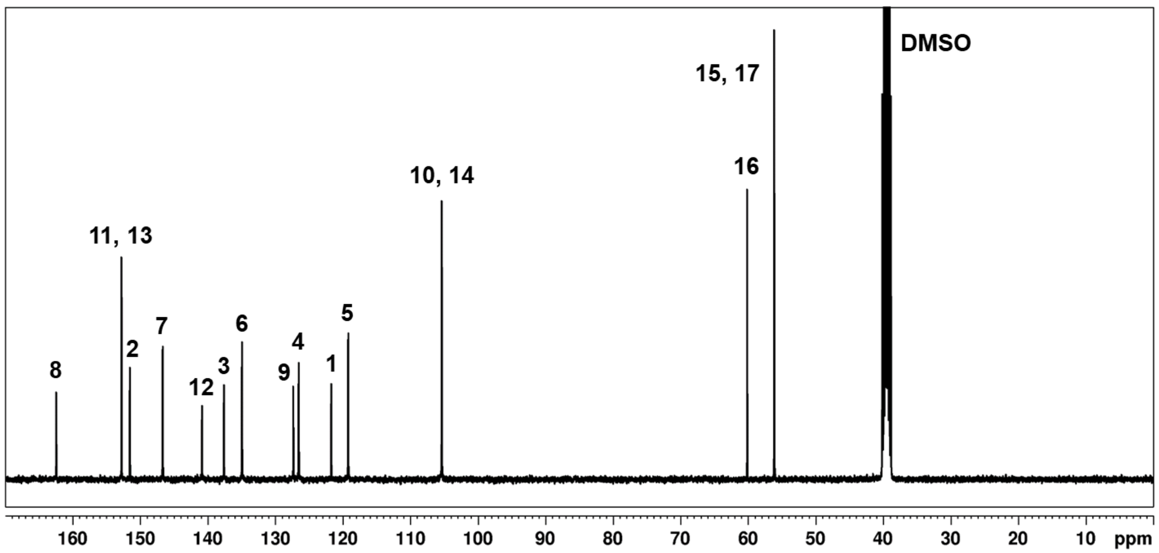

Figure S9: ^{13}C NMR (100 MHz) spectra of **hdz-NO2** in DMSO-d_6 at 25 °C.

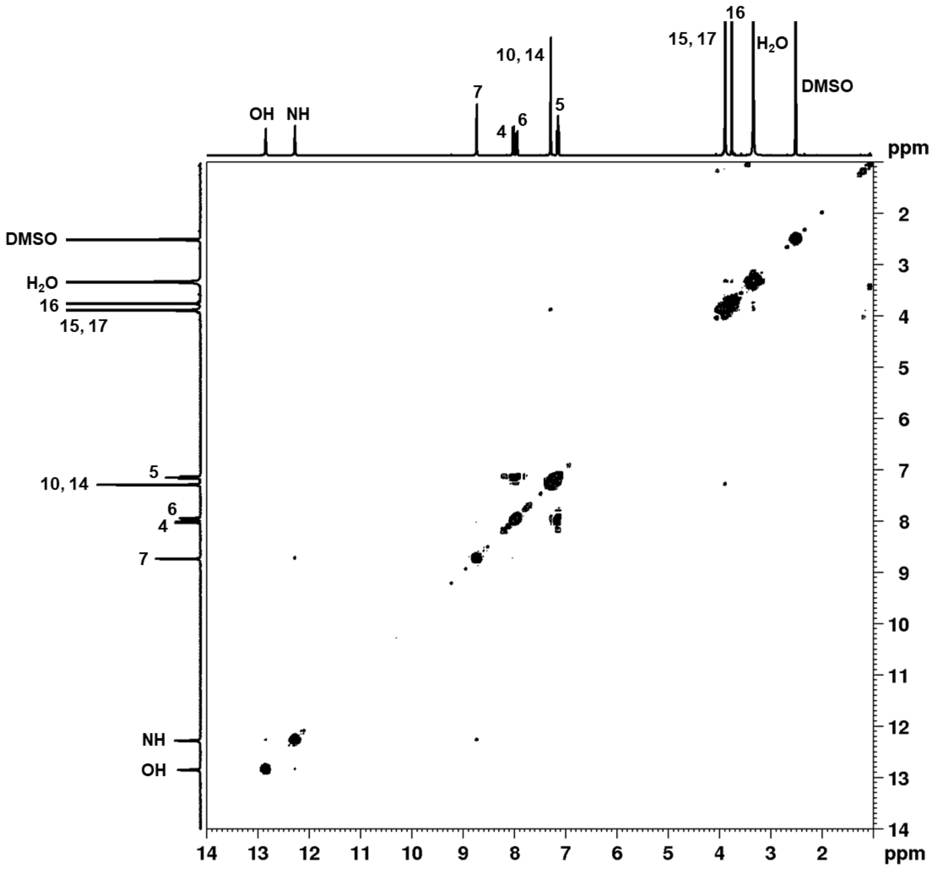

Figure S10: COSY contour map of **hdz-NO2** in DMSO-d_6 at 25 °C.

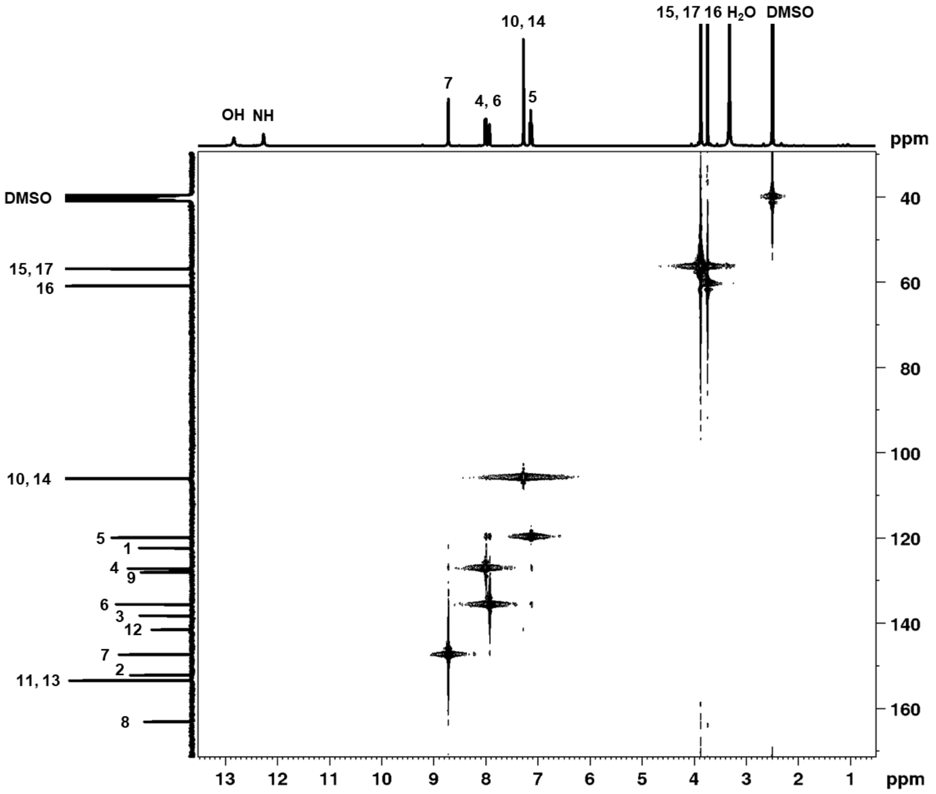

Figure S11: HSQC contour map of **hdz-NO2** in DMSO-d_6 at 25 °C.

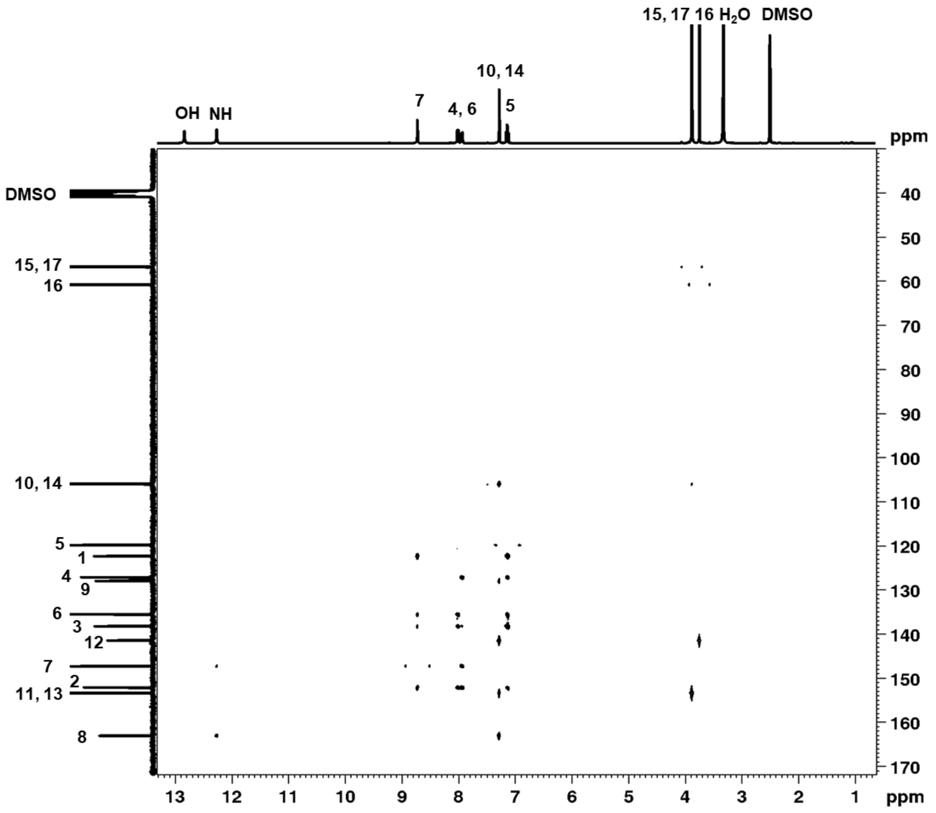

Figure S12: HMBC contour map of **hdz-NO2** in DMSO-d_6 at 25 °C.

Table S5: ^{1}H (400 MHz) and ^{13}C (100 MHz) NMR signal assignments for **hdz-CH$_3$** and **hdz-NO$_2$**, in DMSO-d_6 at 25 °C.

hdz-CH$_3$				hdz-NO$_2$			
C/H	δ ^{13}C (ppm)	δ ^{1}H (ppm)	^{13}C,^{1}H HMBC	C/H	δ ^{13}C (ppm)	δ ^{1}H (ppm)	^{13}C,^{1}H HMBC
**1**	117.22	-	H5 ($^{3}J_{CH}$), H7 ($^{2}J_{CH}$), H18 ($^{4}J_{CH}$)	**1**	121.73	-	H5 ($^{3}J_{CH}$), H7 ($^{2}J_{CH}$)
**2**	155.97	-	H4 ($^{3}J_{CH}$), H5 ($^{4}J_{CH}$), H6 ($^{3}J_{CH}$), H7 ($^{3}J_{CH}$), H18 ($^{3}J_{CH}$)	**2**	151.56	-	H4 ($^{3}J_{CH}$), H5 ($^{4}J_{CH}$), H6 ($^{3}J_{CH}$), H7 ($^{3}J_{CH}$)
**3**	125.03	-	H4 ($^{2}J_{CH}$), H5 ($^{3}J_{CH}$), H7 ($^{4}J_{CH}$), H18 ($^{2}J_{CH}$)	**3**	137.64	-	H4 ($^{2}J_{CH}$), H5 ($^{3}J_{CH}$), H6 ($^{4}J_{CH}$), H7 ($^{4}J_{CH}$)
**4**	132.44	7.22 (d, 1H, $^{3}J_{HH}$ = 7.5 Hz)	H5 ($^{2}J_{CH}$), H6 ($^{3}J_{CH}$), H18 ($^{3}J_{CH}$)	**4**	126.56	8.01 (dd, 1H, $^{3}J_{HH}$ = 7.9 Hz, $^{4}J_{HH}$ = 1.5 Hz)	H5 ($^{2}J_{CH}$), H6 ($^{3}J_{CH}$)
**5**	118.94	6.86 (t, 1H, $^{3}J_{HH}$ = 7.5 Hz)	-	**5**	119.23	7.14 (t, 1H, $^{3}J_{HH}$ = 7.9 Hz)	-
**6**	128.61	7.28 (d, 1H, $^{3}J_{HH}$ = 7.5 Hz)	H4 ($^{3}J_{CH}$), H5 ($^{2}J_{CH}$), H7 ($^{3}J_{CH}$)	**6**	134.95	7.94 (dd, 1H, $^{3}J_{HH}$ = 7.9 Hz, $^{4}J_{HH}$ = 1.5 Hz)	H4 ($^{3}J_{CH}$), H5 ($^{2}J_{CH}$), H7 ($^{3}J_{CH}$)
**7**	149.95	8.58 (s, 1H)	H6 ($^{3}J_{CH}$), NH ($^{3}J_{CH}$)	**7**	146.70	8.74 (s, 1H)	H6 ($^{3}J_{CH}$), NH ($^{3}J_{CH}$)
**8**	162.21	-	H10/H14 ($^{3}J_{CH}$), NH ($^{2}J_{CH}$)	**8**	162.46	-	H10/H14 ($^{3}J_{CH}$), NH ($^{2}J_{CH}$)
**9**	127.65	-	H10/H14 ($^{2}J_{CH}$)	**9**	127.37	-	H10/H14 ($^{2}J_{CH}$)
**10, 14**	105.32	7.27 (s, 2H)	H10/H14 ($^{3}J_{CH}$)*, H15/H17 ($^{4}J_{CH}$)	**10, 14**	105.38	7.28 (s, 2H)	H10/H14 ($^{3}J_{CH}$)*, H15/H17 ($^{4}J_{CH}$)
**11, 13**	152.78	-	H10/H14 ($^{2}J_{CH}$ / $^{4}J_{CH}$), H15/H17 ($^{3}J_{CH}$)	**11, 13**	152.79	-	H10/H14 ($^{2}J_{CH}$ / $^{4}J_{CH}$), H15/H17 ($^{3}J_{CH}$)
**12**	140.70	-	H10/H14 ($^{3}J_{CH}$), H16 ($^{3}J_{CH}$)	**12**	140.87	-	H10/H14 ($^{3}J_{CH}$), H16 ($^{3}J_{CH}$)
**15, 17**	56.16	3.88 (s, 6H)	-	**15, 17**	56.15	3.88 (s, 6H)	-
**16**	60.16	3.74 (s, 3H)	-	**16**	60.16	3.74 (s, 3H)	-
**18**	15.48	2.22 (s, 3H)	H4 ($^{3}J_{CH}$)	**-**	-	-	-
**OH**	-	11.89 (s, 1H)	-	**OH**	-	12.84 (s, 1H)	-
**NH**	-	12.05 (s, 1H)	-	**NH**	-	12.27 (s, 1H)	-

Multiplicity: s = singlet; d = doublet; dd = double of doublets; t = triplet.

*C10 to H14 and C14 to H10

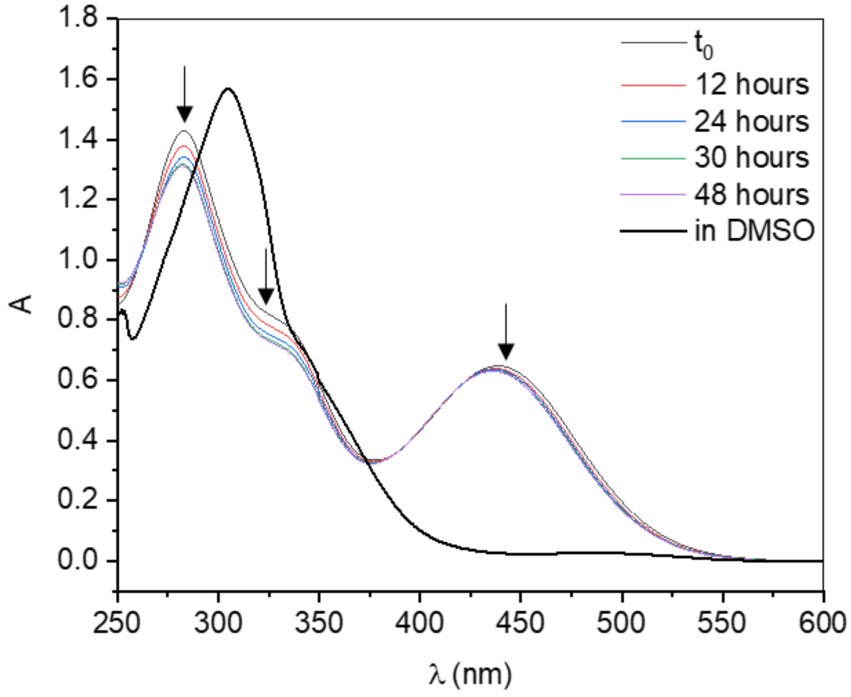

Figure S13: Electronic absorption spectra in selected wavelength regions for a solution of **hdz-NO**$_2$ in 10% DMSO/HEPES mixture (pH 7.4). Experimental conditions: l = 1.0 cm and T = (25.0 ± 0.1) °C. Spectra were measured at t_0 and after 12, 24, 30, and 48 h. Absorption in pure DMSO was added for the sake of comparison.
